# Causal association between autoimmune diseases and risk of breast cancer: A bidirectional Mendelian randomization study

**DOI:** 10.1097/MD.0000000000045283

**Published:** 2025-11-14

**Authors:** Luyao Dai, Danxiang Chen, Xinlin Li, Qikuan He, Jing Jiang, Xujun Li

**Affiliations:** a Department of Oncology, Ningbo No. 2 Hospital, Ningbo, Zhejiang, PR China; b Department of Breast Surgery, Ningbo No. 2 Hospital, Ningbo, Zhejiang, PR China.

**Keywords:** autoimmune diseases, breast cancer, GWAS, Mendelian randomization

## Abstract

The potential causal relationship between autoimmune diseases (AIDs) and breast cancer (BC) remains a topic of debate. In this study, we conducted Mendelian randomization (MR) analysis between common AIDs and BC using publicly available genome-wide association studies data from the MR Base platform. Initially, we conducted a two-sample MR analysis of 10 AIDs and BC using the inverse variance weighted method. Subsequently, a series of sensitivity analyses were performed to validate the robustness of our findings. Lastly, we utilized the reverse MR analysis to evaluate the potential for reverse causality. Among the 10 types of AID studied, 3 were found to be associated with an elevated risk of BC. These include ankylosing spondylitis (odds ratio [OR]: 1.21; 95% confidence interval [CI]: 1.04–1.41; *P* = .013), inflammatory bowel disease (OR: 1.05; 95% CI: 1.01–1.09; *P* = .012), and rheumatoid arthritis (OR: 1.04; 95% CI: 1.01–1.07; *P* = .023). Additionally, BC was found to be linked with a heightened risk of inflammatory bowel disease (OR: 1.112; 95% CI: 1.022–1.210; *P* = .014). Our study’s findings support a bidirectional causal relationship between AIDs and BC, offering novel insights into the developmental mechanisms underlying the interaction between AIDs and BC.

## 1. Introduction

Cancer immunotherapy is a treatment approach that harnesses the patient’s immune system to fight against cancer.^[1]^ It can stimulate and enhance the immune response against tumors, which may also lead to the development of autoimmune diseases (AIDs). Over the years, the relationship between AIDs and cancer has gained attention, with several studies suggesting that individuals with AID have a higher risk of developing tumors compared to the general population. For example, patients with systemic lupus erythematosus (SLE) and rheumatoid arthritis (RA) have an increased risk of lymphoma,^[2,3]^ while long-term ulcerative colitis (UC) and Crohn disease (CD) of the colon are associated with a significantly higher risk of colorectal cancer.^[4]^

In 2020, breast cancer (BC) became the most common cancer worldwide.^[5]^ Observational studies have indicated an association between certain AIDs, such as autoimmune thyroiditis, SLE, and multiple sclerosis (MS), and an increased risk of BC.^[6]^ This suggests that immune dysfunction and immunosuppressive therapy may contribute to BC susceptibility. However, the causal relationship between AIDs and BC has not been definitively established, as traditional observational studies have limitations such as confounding bias and reverse causality.^[7]^ Therefore, it is important to investigate the true causal relationship between AIDs and BC, as it may shed light on the underlying etiology of BC.

Mendelian randomization (MR) is a method that utilizes genetic variations as instrumental variables (IVs) to infer causality between exposure and outcome associations. Compared to traditional observational studies, MR provides a more reliable and accurate approach by minimizing potential biases.^[8]^ Genetic variations are randomly inherited during conception and are not influenced by environmental or lifestyle factors, nor are they affected by disease development.^[9]^ In recent years, MR methods have been widely employed to assess causal relationships in various complex diseases, including AIDs.^[10–13]^ Additionally, genome-wide association studies (GWASs) provide a large-scale dataset of genetic variants, serving as IVs for MR analysis. Utilizing these datasets instead of individual-level data improves the ability to evaluate causal effects.

While one MR study found no causal link between AIDs and BC risk in the European population, this study only investigated 5 types of AID.^[14]^ In our study, we aimed to explore the bidirectional causal relationship between a broader range of AIDs and BC. We conducted a comprehensive MR analysis using GWASs data on 10 AIDs, including ankylosing spondylitis (AS), celiac disease (CeD), inflammatory bowel disease (IBD), MS, primary biliary cholangitis (PBC), psoriasis (PsO), RA, SLE, type 1 diabetes (T1D), and myasthenia gravis (MG). Our analysis included both positive MR analysis, considering AIDs as the exposure and BC as the outcome, and reverse MR analysis, considering BC as the exposure and AIDs as the outcome.

## 2. Methods

### 2.1. Study design

**Figure 1.**
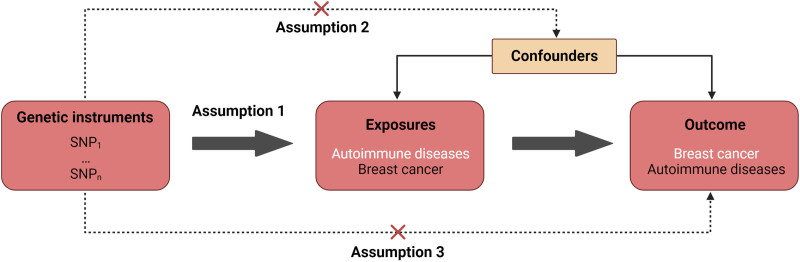
Study design overview. SNP = single nucleotide polymorphism.

Figure 1 provides an overview of the study design. This study aims to assess the causal relationship between AIDs and BC risk using a two-sample MR approach. In this study, single nucleotide polymorphisms (SNPs) identified through large-scale GWASs were utilized as IVs to represent exposures. The MR analysis is based on 3 important assumptions. Firstly, the association assumption states that the genetic variation used as IVs should be strongly associated with the exposure factor. Secondly, the independence assumption suggests that the genetic variation used is not associated with any confounding factors. Lastly, the exclusion restriction assumption posits that the selected genetic variant can only affect the risk of the outcome through the exposure factors and not through other pathways.

### 2.2. Data sources and selection of genetic instrument

The MR Base database (http://www.mrbase.org/) collects a wealth of aggregated statistics from hundreds of GWASs. All datasets are publicly available and can be downloaded as open-source files, thus no additional ethical review is required. To minimize potential bias caused by population stratification, only subjects with a European ancestry background were selected. AID-associated SNPs were used as IVs at the genome-wide significance level (*P* < 5 × 10^−8^) to satisfy the first assumption. Additionally, to account for the effects of linkage disequilibrium and ensure independence of each IV, a statistical significance threshold of “*r*^2^ < 0.001 and clumping distance > 10,000” was set. Using publicly available GWASs, 10 types of AID were obtained (Table 1), including AS, CeD, IBD, MS, PBC, PsO, RA, SLE, T1D, and MG. As an outcome indicator, the GWASs aggregated data for BC were obtained from the Breast Cancer Association Consortium, which was published by Michailidou K et al in 2017. The sample size included 32,498 cases, with 14,910 cases and 17,588 cases in the control group.

**Table 1:** Genome-wide association studies samples used in this study.

Trait	First author	Consortium	Cases, n	Controls, n	Year	PubMed ID
Ankylosing spondylitis	Cortes A	NA	9069	1550	2013	23749187
Celiac disease	Trynka G	NA	11,812	229	2011	22057235
Inflammatory bowel disease	Liu	IIBDGC	31,665	33,977	2015	26192919
Multiple sclerosis	Beecham AH	IMSGC	14,498	24,091	2013	24076602
Primary biliary cholangitis	Cordell HJ	NA	2764	10,475	2015	26394269
Psoriasis	Tsoi LC	NA	10,588	22,806	2012	23143594
Rheumatoid arthritis	Ha E	NA	14,361	43,923	2020	33310728
Systemic lupus erythematosus	Bentham J	NA	5201	9066	2015	26502338
Type 1 diabetes	Forgetta V	NA	9266	15,574	2020	32005708
Myasthenia gravis	Chia R	NA	1873	36,370	2022	35074870
Breast cancer	Michailidou K	BCAC	14,910	17,588	2017	29059683

BCAC = Breast Cancer Association Consortium.

### 2.3. Statistical analysis

Conventional random effects inverse variance weighted (IVW) analysis was used for MR analysis. The IVW method combines meta-analysis with Wald ratios obtained from different SNPs to provide estimates of causality between the exposure and outcome.^[15,16]^ The estimate of causality between AIDs and BC was expressed as an odds ratio (OR) with a 95% confidence interval (CI). A *P*-value <.05 indicated a statistically significant difference. Heterogeneity refers to the variability of each SNP’s causal estimate. Low heterogeneity indicates higher reliability of the MR estimation. IVW and MR-Egger methods were used to quantify the heterogeneity effect among the IVs, expressed by Cochran *Q* statistic. The MR-Egger method is a weighted linear regression that can be used to test for horizontal pleiotropy. When the intercept value is close to 0 and *P*-value > .05, it indicates the absence of horizontal pleiotropy. In such cases, the IVW method is considered reliable. The “leave-one-out” method involves calculating MR results with each SNP removed one at a time. If the results change significantly after removing a particular SNP, it indicates that MR results are sensitive to that SNP. Therefore, the “leave-one-out” method was used to assess the impact of each SNP on the MR analysis results. The F statistic was used to evaluate the strength of the extracted IVs, with an F value >10 indicating a low likelihood of weak instrument bias. All MR analysis was performed using the MR Base platform.

## 3. Results

### 3.1. Selection of IVs

Details of the data sets selected for this study were shown in Table 1. Each disease used a specific number of SNPs as IVs, and more information about individual SNPs can be found in Table S1 and S2, Supplemental Digital Content, https://links.lww.com/MD/Q644. The F statistic for all IVs ranged from 29.52 to 1487.90, which was greater than the commonly chosen value of 10, indicating that the IVs used had good strength (Table S1 and S2, Supplemental Digital Content, https://links.lww.com/MD/Q644).

### 3.2. MR results

**Figure 2.**
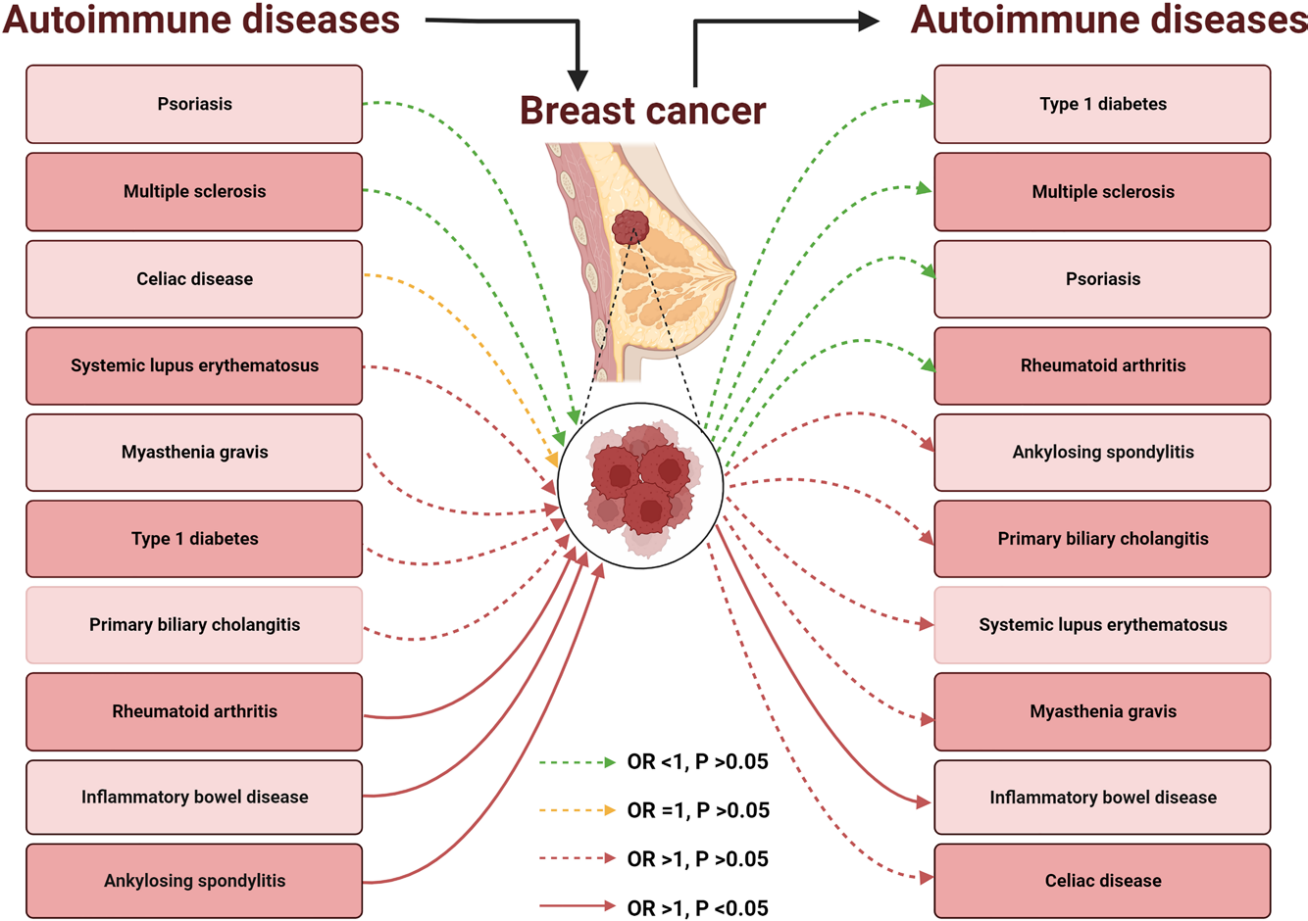
The bidirectional causal relationship between autoimmune diseases and breast cancer. OR = odds ratio.

The IVW method provided evidence supporting a bidirectional causal relationship between certain AIDs and BC, as shown in Table 2. We visually illustrated this cause-and-effect relationship in Figure 2. Among the 10 AIDs, 3 showed a positive correlation with an increased risk of BC, and the correlation was statistically significant (*P* < .05). Specifically, AS (OR: 1.21; 95% CI: 1.04–1.41; *P* = .013), IBD (OR: 1.05; 95% CI: 1.01–1.09; *P* = .012), and RA (OR: 1.04; 95% CI: 1.01–1.07; *P* = .023) were associated with a higher risk of BC. No causal relationship was found between the other 7 types of AIDs and BC. In reverse MR analysis (Table S3, Supplemental Digital Content, https://links.lww.com/MD/Q644), BC was only associated with a higher risk of IBD (OR: 1.112; 95% CI: 1.022–1.210; *P* = .014).

**Table 2:** Mendelian randomization estimates of the associations between autoimmune diseases and breast cancer using inverse variance weighted.

Exposure	b	SE	OR	95% CI	*P* value
Ankylosing spondylitis	0.193	0.077	1.213	1.043–1.410	.013
Celiac disease	−0.000	0.011	1.000	0.978–1.022	.973
Inflammatory bowel disease	0.047	0.019	1.048	1.010–1.088	.012
Multiple sclerosis	−0.008	0.024	0.992	0.946–1.040	.729
Primary biliary cholangitis	0.010	0.017	1.010	0.977–1.044	.546
Psoriasis	−0.006	0.005	0.994	0.984–1.004	.221
Rheumatoid arthritis	0.039	0.017	1.040	1.006–1.075	.023
Systemic lupus erythematosus	0.002	0.012	1.002	0.979–1.026	.890
Type 1 diabetes	0.008	0.012	1.008	0.985–1.032	.513
Myasthenia gravis	0.010	0.045	1.010	0.925–1.103	.829

### 3.3. Sensitivity test

Sensitivity analysis was conducted through 3 aspects: Cochran *Q* test, MR Egger intercept analysis, and “leave-one-out” test. Cochran *Q* test revealed evidence of heterogeneity among IVs estimates based on individual variation for IBD, MS, PsO, RA, and MG (*P* < .05), no heterogeneity was observed among IV estimates for the other 5 types of AID (Table 3). Pleiotropy occurs when the IV affects the outcome through factors other than the exposure. In such cases, even if the MR results were significant, a causal relationship between exposure and outcome cannot be established. Importantly, all AID-level pleiotropy tests yielded *P*-values >.05, indicating the absence of confounding factors in this study. Moreover, based on the results of the “leave-one-out” method, no single SNP played a significant role in causal inference for the 3 AIDs of AS, IBD, and RA (Figs. S1–S3, Supplemental Digital Content, https://links.lww.com/MD/Q644). Sensitivity analysis of reverse MR (Table S4, Supplemental Digital Content, https://links.lww.com/MD/Q644) revealed no pleiotropy or heterogeneity in the IVs of BC. The results of the “leave one method” showed that the significant causal relationship between BC and IBD was not driven by a single SNP (Fig. S4, Supplemental Digital Content, https://links.lww.com/MD/Q644).

**Table 3:** Sensitivity analyses of the associations between different autoimmune diseases and breast cancer.

Exposure	Heterogeneity				Horizontal pleiotropy	
	MR Egger		IVW			
	Q statistic	*P*	Q statistic	*P*	Intercept	*P*
Ankylosing spondylitis	16.230	.757	17.970	.708	0.009	.201
Celiac disease	36.280	.409	36.320	.454	0.001	.838
Inflammatory bowel disease	163.20	.019	165.00	.018	0.006	.238
Multiple sclerosis	55.340	.052	56.840	.037	−0.011	.148
Primary biliary cholangitis	29.640	.100	32.220	.074	−0.023	.191
Psoriasis	66.950	.029	67.730	.032	0.005	.465
Rheumatoid arthritis	112.10	.013	112.30	.015	−0.002	.712
Systemic lupus erythematosus	44.290	.191	46.440	.164	−0.012	.189
Type 1 diabetes	47.820	.090	48.080	.105	0.003	.656
Myasthenia gravis	9.979	.041	10.900	.053	−0.025	.577

IVW = inverse variance weighted, MR = Mendelian randomization.

## 4. Discussion

To explore the mechanisms of disease, we usually classify the association between observed risk factors and endpoint outcomes as either causal or noncausal. MR is a potentially powerful method to enhance causal inference in these associations. Given the inconsistent results from observational research on the risk of AIDs and BC, as well as potential methodological limitations, we conducted a bidirectional MR analysis of 2 samples to investigate the causal relationship between 10 common AIDs (AS, CeD, IBD, MS, PBC, PsO, RA, SLE, T1D, and MG) and BC. Based on the analysis results, we determined that there was a causal relationship between AS, IBD, RA and the risks of BC, and we also found a reverse causal relationship between IBD and BC. Nevertheless, the relationship between these 3 types of AID and BC has been controversial.

A retrospective cohort study suggested that AS was not associated with an increased risk of BC diagnosis.^[17]^ However, Feng et al found that high levels of interleukin (IL)-17 secreted in AS may act as a tumor-promoting factor for BC.^[18]^ From a pathophysiological perspective, the biomarker IL-17 may be a common therapeutic target for both AS and BC.

IBD has been reported to have no significant effect on the risk of developing BC.^[19]^ However, a retrospective cohort study conducted by Tsai et al showed that the risk of developing BC was proportional to the frequency of hospitalization in patients with IBD. Compared to the control group, IBD patients who required more than 2 hospital admissions per year had an approximately 10-fold increased risk of developing BC.^[20]^ IBD includes UC and CD. Zhou et al pointed out in a study that CD was an independent risk factor for the onset of BC, and CD patients had a higher risk of developing BC than non-CD patients. Using bioinformatics tools, they also discovered that both CD and BC were associated with the IL-17 and NF-κB signaling pathways.^[21]^ Another prospective study noted an increase in the standardized incidence of BC in both UC and CD populations.^[22]^ BC resistance protein is an efflux transporter that protects intestinal cells from toxic compounds. This transporter is downregulated in patients with active UC. Therefore, the authors suggested that the inflammatory process of IBD was the cause of the reduced BC resistance protein levels, which may be related to the pathogenesis of BC. In this study, we also found a high risk of IBD in patients with BC. However, little has been reported about the risk of developing IBD in BC patients. Dedousis et al proposed in a study that people with BC had a higher prevalence of IBD compared to the general population.^[23]^ Another study showed that one of the metastatic manifestations of invasive lobular BC was IBD, although this was a very rare case.^[24]^

A meta-analysis showed that patients with RA had a reduced risk of BC (OR = 0.84, 95% CI: 0.79–0.90).^[25]^ This may be due to increased immune surveillance resulting from improved overall immunomodulatory function. Another meta-analysis showed that while RA patients did not have an increased risk of BC, nonwhite RA patients had an increased risk of BC in a subgroup analysis.^[26]^ Additionally, a cohort study in a Taiwanese population showed an increased risk of BC in patients with RA (OR = 1.21, 95% CI: 1.19–1.23).^[27]^

The exact mechanisms underlying the interaction between AIDs and cancer are not well understood. There are several potential mechanisms that may explain the higher cancer risk associated with AIDs. These include autoimmune-induced chronic inflammation and immunosuppression. Patients with AIDs often have elevated levels of pro-inflammatory cytokines such as tumor necrosis factor-α, IL-1, IL-6, and chemokines in their bloodstream. However, anti-inflammatory cytokines like IL-10 and transforming growth factor-β are upregulated to protect normal tissues from the long-term effects of pro-inflammatory cytokines.^[28]^ This inflammatory environment promotes the initiation and early growth of cancer, thereby increasing the cancer risk. Additionally, immune disorders in AID patients may make them more susceptible to certain types of cancer. The long-term use of immunosuppressive therapies in AID patients leads to immune dysfunction, which impairs the body’s ability to clear cancer-causing infections.^[29]^ Studies have shown that the use of certain immunosuppressive drugs such like thiopurine,^[30]^ methotrexate,^[31]^ and cyclophosphamide^[32]^ can increase the risk of developing malignant tumors.

The connection between AIDS and cancer is bidirectional, and this discovery also has significant therapeutic implications. Chronic inflammation caused by AIDs or its treatment can contribute to the initiation and progression of cancer. Conversely, antitumor immunotherapy can stimulate and enhance the host’s immune response against tumor cells, which may also lead to the development of autoimmunity.^[33,34]^ Additionally, radiation and chemotherapy used in cancer treatment can cause massive necrosis of cancer cells and surrounding tissues, which in turn triggers an inflammatory response. This therapeutic inflammation can both promote tumor growth and metastasis, as well as enhance the presentation of tumor antigens, leading to an antitumor immune response.^[35]^ Endocrine therapies such as tamoxifen or aromatase inhibitors may regulate immune function and inflammatory pathways, but their direct impact on autoimmunity remains unclear. Therefore, when choosing treatment strategies, clinicians should be aware of the potential bidirectional interaction between AIDs and BC, especially in patients with comorbidities.

Although the present MR analysis did not directly assess survival outcomes, emerging evidence from observational studies suggests that comorbid AIDs may influence BC prognosis. For instance, patients with RA or SLE often receive immunosuppressive therapies, which could potentially impair antitumor immunity and thereby affect cancer progression or recurrence. Conversely, immune activation in certain AIDs might enhance immune surveillance and improve response to immunotherapy. A recent cohort study by Dedousis et al^[23]^ reported that the presence of AIDs diagnosis was associated with a lower survival period in patients with stage I–III BC and an improved survival period in patients with stage IV BC. However, the results remain inconsistent, likely due to heterogeneity in AID types, treatments, and BC subtypes. Future prospective studies and MR analyses incorporating survival data are warranted to elucidate the causal role of AIDs in BC prognosis and to inform personalized treatment strategies for this patient subgroup.

In a previous study, Lu et al investigated the interaction between 5 types of AID (Graves’ disease, Sjogren syndrome, PsO, CD, and systemic scleroderma) and BC using MR analysis.^[14]^ However, our study differs from theirs in 2 main aspects. Firstly, our research provides a more comprehensive analysis of AIDs. Unlike the analysis of 5 diseases mentioned above, we comprehensively analyzed 10 common AIDs. This allows us to evaluate BC that is causally associated with multiple AIDs. Secondly, our study implemented more stringent quality control procedures in selecting IVs. Lu et al used a relatively loose *P*-value threshold (*P* < 5 × 10^−6^) and a linkage imbalance correlation coefficient *r*^2^ (*r*^2^ < 0.01) to select eligible IVs. In contrast, our study chose the “gold standard” of *P*-value threshold (*P* < 5 × 10^−8^) and removed more SNPs with linkage imbalance (*r*^2^ < 0.001). It is worth mentioning that, compared to previous observational studies, our study also benefits from the inherent advantages of all MR analyses, such as minimizing the risk of confounding and reverse causal relationships.

Nevertheless, our study has some limitations. A major limitation is the lack of IVs for many AIDs, which limits our selection of AIDs and prevents us from examining more bidirectional associations between AIDs and BC. Secondly, the Cochran *Q* test in the forward MR analysis showed significant heterogeneity. However, this did not affect the results of the IVW method, and the results of our MR analysis remained reliable. Additionally, the participants in our study were of European descent. While this limits population bias, it may also limit the generalizability of our findings to other ancestral populations. Finally, it is worth noting that our study did not differentiate between molecular subtypes of BC (e.g., luminal A/B, HER2-enriched, and triple-negative) due to the lack of subtype-stratified GWASs summary data. Future studies with access to subtype-specific genetic instruments may provide deeper insights into the heterogeneous relationships between AIDs and different BC subtypes.

In summary, our findings support the bidirectional causal relationship between AIDs and BC. This suggests that, on the one hand, AIDs may increase the risk of BC, and on the other hand, BC may also trigger autoimmunity through different mechanisms. Future research should aim to elucidate the underlying mechanisms of the association between AIDs and BC, and explore new strategies for the prevention and treatment of both diseases.

## 5. Conclusions

This comprehensive bidirectional MR analysis provides insights into the causal relationship between AIDs and BC. Our analysis reveals that AIDs such as AS, IBD, and RA can increase the risk of developing BC. Conversely, BC is also associated with an increased risk of developing IBD. The specific mechanisms underlying the interaction between AIDs and BC require further investigation. We hope that future research will shed light on these mechanisms, enabling us to improve the detection, prevention, and treatment of both AIDs and BC.

## Acknowledgments

We thank the creators of MR Base and the providers of GWASs data.

## Author contributions

**Conceptualization:** Luyao Dai.

**Data curation:** Danxiang Chen.

**Formal analysis:** Qikuan He.

**Investigation:** Xinlin Li.

**Methodology:** Jing Jiang.

**Validation:** Xujun Li.

**Writing – original draft:** Luyao Dai.

**Writing – review & editing:** Qikuan He.

## Supplementary Material


