# Supplementary material for: Causal association between autoimmune diseases and risk of breast cancer: A bidirectional Mendelian randomization study
Source: Medicine (Baltimore). 2025 Nov 14;104(46):e45283. doi: 10.1097/MD.0000000000045283 (PMC12622599; doi:10.1097/MD.0000000000045283)
Supplement: Supplementary file 1 [file medi-104-e45283-s001.docx]

**Table S1** Details of all SNPs for 10 autoimmune diseases

|  | **Exposure** | **SNP** | **Effect allele** | **Other allele** | **Sample size** | **Beta** | **SE** | **R2** | **F** |
| --- | --- | --- | --- | --- | --- | --- | --- | --- | --- |
| 1 | Ankylosing spondylitis | rs1041926 | A | G | 10619 | -0.0748291 | 0.0116918 | 0.003842578 | 40.95 |
| 2 | Ankylosing spondylitis | rs11190133 | T | C | 10619 | -0.0338671 | 0.00449401 | 0.005319709 | 56.78 |
| 3 | Ankylosing spondylitis | rs11209026 | A | G | 10619 | -0.103584 | 0.00954481 | 0.01096925 | 117.75 |
| 4 | Ankylosing spondylitis | rs1128905 | C | T | 10619 | -0.0237165 | 0.00409459 | 0.003149393 | 33.54 |
| 5 | Ankylosing spondylitis | rs11624293 | C | T | 10619 | 0.0428679 | 0.00669161 | 0.003849855 | 41.03 |
| 6 | Ankylosing spondylitis | rs1250550 | A | C | 10619 | -0.026036 | 0.00430465 | 0.003433173 | 36.58 |
| 7 | Ankylosing spondylitis | rs12615545 | C | T | 10619 | 0.0254728 | 0.00417273 | 0.003497098 | 37.26 |
| 8 | Ankylosing spondylitis | rs1801274 | G | A | 10619 | 0.0253178 | 0.00417687 | 0.003447993 | 36.73 |
| 9 | Ankylosing spondylitis | rs2517655 | T | C | 10619 | 0.0882856 | 0.00465575 | 0.032753228 | 359.52 |
| 10 | Ankylosing spondylitis | rs2531875 | T | G | 10619 | -0.0273233 | 0.00424463 | 0.003886974 | 41.43 |
| 11 | Ankylosing spondylitis | rs2596501 | T | C | 10619 | -0.152336 | 0.00419401 | 0.110510462 | 1,319.06 |
| 12 | Ankylosing spondylitis | rs27529 | G | A | 10619 | -0.0620354 | 0.00429859 | 0.019235707 | 208.23 |
| 13 | Ankylosing spondylitis | rs2836883 | A | G | 10619 | -0.0396768 | 0.00474798 | 0.00653319 | 69.82 |
| 14 | Ankylosing spondylitis | rs35164067 | A | G | 10619 | -0.031078 | 0.00495021 | 0.003697995 | 39.41 |
| 15 | Ankylosing spondylitis | rs4129267 | T | C | 10619 | -0.0307685 | 0.00422605 | 0.004967035 | 53.00 |
| 16 | Ankylosing spondylitis | rs41299637 | G | T | 10619 | -0.0390527 | 0.00491011 | 0.005921839 | 63.25 |
| 17 | Ankylosing spondylitis | rs4672505 | G | A | 10619 | -0.0597772 | 0.00415105 | 0.019154559 | 207.34 |
| 18 | Ankylosing spondylitis | rs4676410 | A | G | 10619 | 0.0281014 | 0.00490215 | 0.003085015 | 32.85 |
| 19 | Ankylosing spondylitis | rs6556416 | C | A | 10619 | 0.0252152 | 0.0046002 | 0.002821375 | 30.04 |
| 20 | Ankylosing spondylitis | rs6600247 | C | T | 10619 | 0.0328332 | 0.00415088 | 0.005857484 | 62.56 |
| 21 | Ankylosing spondylitis | rs7191548 | C | T | 10619 | 0.0249853 | 0.00428493 | 0.003191609 | 33.99 |
| 22 | Ankylosing spondylitis | rs743479 | T | C | 10619 | -0.0234235 | 0.00417581 | 0.002954293 | 31.46 |
| 23 | Ankylosing spondylitis | rs9901869 | A | G | 10619 | 0.0319036 | 0.00408853 | 0.005701357 | 60.88 |
| 1 | Celiac disease | rs1018326 | C | T | 12041 | 0.151862 | 0.0185859 | 0.005514005 | 66.75 |
| 2 | Celiac disease | rs1050976 | T | C | 12041 | -0.111043 | 0.0184718 | 0.002992264 | 36.13 |
| 3 | Celiac disease | rs10790269 | T | C | 12041 | 0.157239 | 0.0239751 | 0.003559496 | 43.01 |
| 4 | Celiac disease | rs10947460 | A | G | 12041 | -0.12545 | 0.0227727 | 0.00251395 | 30.34 |
| 5 | Celiac disease | rs11801183 | T | C | 12041 | -0.138343 | 0.0245229 | 0.0026361 | 31.82 |
| 6 | Celiac disease | rs11851414 | C | T | 12041 | 0.120446 | 0.0220512 | 0.002471627 | 29.83 |
| 7 | Celiac disease | rs11875687 | C | T | 12041 | 0.159565 | 0.0250579 | 0.003356319 | 40.54 |
| 8 | Celiac disease | rs12068671 | C | T | 12041 | -0.156654 | 0.0244153 | 0.003407328 | 41.16 |
| 9 | Celiac disease | rs1250552 | G | A | 12041 | -0.154784 | 0.0185777 | 0.005732039 | 69.41 |
| 10 | Celiac disease | rs13003464 | G | A | 12041 | 0.153579 | 0.0188934 | 0.005457631 | 66.06 |
| 11 | Celiac disease | rs13132308 | G | A | 12041 | -0.34899 | 0.0269131 | 0.013772509 | 168.12 |
| 12 | Celiac disease | rs13195040 | G | A | 12041 | 1.12005 | 0.0291168 | 0.109442711 | 1,479.50 |
| 13 | Celiac disease | rs1323292 | A | G | 12041 | 0.261754 | 0.025293 | 0.008816123 | 107.08 |
| 14 | Celiac disease | rs1378938 | C | T | 12041 | -0.117783 | 0.0204027 | 0.002760114 | 33.32 |
| 15 | Celiac disease | rs1431403 | C | T | 12041 | 0.64815 | 0.0199541 | 0.0805648 | 1,054.91 |
| 16 | Celiac disease | rs17264332 | G | A | 12041 | 0.250759 | 0.0220259 | 0.0106496 | 129.59 |
| 17 | Celiac disease | rs182429 | G | A | 12041 | -0.150143 | 0.0186583 | 0.00534902 | 64.74 |
| 18 | Celiac disease | rs1893592 | C | A | 12041 | -0.123864 | 0.0208749 | 0.002915485 | 35.20 |
| 19 | Celiac disease | rs1980422 | T | C | 12041 | -0.172271 | 0.0215795 | 0.005264856 | 63.72 |
| 20 | Celiac disease | rs2030519 | A | G | 12041 | 0.27826 | 0.0188629 | 0.017751837 | 217.58 |
| 21 | Celiac disease | rs2097282 | T | C | 12041 | -0.183987 | 0.0197345 | 0.007166972 | 86.91 |
| 22 | Celiac disease | rs2269423 | C | A | 12041 | 0.925079 | 0.0240484 | 0.109442203 | 1,479.49 |
| 23 | Celiac disease | rs2499714 | T | C | 12041 | 0.188966 | 0.0305353 | 0.003170453 | 38.29 |
| 24 | Celiac disease | rs4445406 | C | T | 12041 | -0.136049 | 0.0197343 | 0.003931641 | 47.52 |
| 25 | Celiac disease | rs4821124 | C | T | 12041 | 0.151003 | 0.0230512 | 0.003551211 | 42.91 |
| 26 | Celiac disease | rs55743914 | T | C | 12041 | 0.187309 | 0.021237 | 0.006419063 | 77.78 |
| 27 | Celiac disease | rs61579022 | A | G | 12041 | 0.107957 | 0.0188338 | 0.00272132 | 32.85 |
| 28 | Celiac disease | rs61907765 | T | C | 12041 | 0.161268 | 0.0221632 | 0.004377876 | 52.94 |
| 29 | Celiac disease | rs6498114 | T | G | 12041 | -0.131028 | 0.0211512 | 0.003176974 | 38.37 |
| 30 | Celiac disease | rs6715106 | G | A | 12041 | -0.237369 | 0.0412053 | 0.002748426 | 33.18 |
| 31 | Celiac disease | rs7104791 | C | T | 12041 | -0.14842 | 0.0221049 | 0.003730115 | 45.07 |
| 32 | Celiac disease | rs744254 | A | G | 12041 | 0.116421 | 0.0210172 | 0.002541822 | 30.68 |
| 33 | Celiac disease | rs76830965 | A | C | 12041 | 0.307485 | 0.0283999 | 0.009641489 | 117.20 |
| 34 | Celiac disease | rs79758729 | G | A | 12041 | 0.162969 | 0.0290899 | 0.002599755 | 31.38 |
| 35 | Celiac disease | rs9258302 | C | T | 12041 | -0.357818 | 0.0387501 | 0.007031559 | 85.25 |
| 36 | Celiac disease | rs9268303 | A | G | 12041 | -0.638659 | 0.0272424 | 0.043651673 | 549.51 |
| 37 | Celiac disease | rs990171 | C | A | 12041 | -0.178146 | 0.0215135 | 0.00566241 | 68.56 |
| 1 | Inflammatory bowel disease | rs10142466 | G | A | 65642 | -0.0580054 | 0.010146 | 0.000497679 | 32.68 |
| 2 | Inflammatory bowel disease | rs10758669 | A | C | 65642 | -0.148762 | 0.0102138 | 0.003221264 | 212.13 |
| 3 | Inflammatory bowel disease | rs10761659 | G | A | 65642 | 0.153811 | 0.0100347 | 0.003566421 | 234.94 |
| 4 | Inflammatory bowel disease | rs10800309 | G | A | 65642 | -0.132111 | 0.010405 | 0.002449891 | 161.21 |
| 5 | Inflammatory bowel disease | rs10956252 | G | C | 65642 | 0.0834795 | 0.0101714 | 0.001025111 | 67.36 |
| 6 | Inflammatory bowel disease | rs11152949 | G | A | 65642 | 0.105057 | 0.0106717 | 0.001474213 | 96.91 |
| 7 | Inflammatory bowel disease | rs11185982 | C | T | 65642 | -0.077882 | 0.0137863 | 0.000485944 | 31.91 |
| 8 | Inflammatory bowel disease | rs11230563 | T | C | 65642 | -0.081194 | 0.0105845 | 0.000895646 | 58.84 |
| 9 | Inflammatory bowel disease | rs11236797 | A | C | 65642 | 0.150864 | 0.00996698 | 0.003478156 | 229.10 |
| 10 | Inflammatory bowel disease | rs11641016 | G | C | 65642 | -0.111288 | 0.0133908 | 0.001051103 | 69.07 |
| 11 | Inflammatory bowel disease | rs11677953 | A | G | 65642 | 0.0790564 | 0.0100145 | 0.000948466 | 62.32 |
| 12 | Inflammatory bowel disease | rs11691685 | G | A | 65642 | -0.122467 | 0.0187977 | 0.000646199 | 42.44 |
| 13 | Inflammatory bowel disease | rs11713774 | C | T | 65642 | 0.0942858 | 0.0142704 | 0.000664584 | 43.65 |
| 14 | Inflammatory bowel disease | rs11758694 | A | T | 65642 | 0.107782 | 0.0160257 | 0.000688617 | 45.23 |
| 15 | Inflammatory bowel disease | rs11768997 | T | G | 65642 | 0.155265 | 0.0180166 | 0.00113013 | 74.27 |
| 16 | Inflammatory bowel disease | rs11793497 | G | A | 65642 | 0.156206 | 0.0100484 | 0.003667954 | 241.65 |
| 17 | Inflammatory bowel disease | rs1182188 | C | T | 65642 | -0.0659028 | 0.0108086 | 0.000566032 | 37.18 |
| 18 | Inflammatory bowel disease | rs12103 | C | T | 65642 | -0.0867354 | 0.0130755 | 0.00066989 | 44.00 |
| 19 | Inflammatory bowel disease | rs12411259 | A | G | 65642 | 0.0669054 | 0.0115117 | 0.000514326 | 33.78 |
| 20 | Inflammatory bowel disease | rs1250566 | A | G | 65642 | -0.100894 | 0.0110038 | 0.001279109 | 84.07 |
| 21 | Inflammatory bowel disease | rs12585310 | A | G | 65642 | 0.0706485 | 0.0107636 | 0.000655879 | 43.08 |
| 22 | Inflammatory bowel disease | rs1267499 | C | T | 65642 | 0.0821443 | 0.0125131 | 0.000656082 | 43.09 |
| 23 | Inflammatory bowel disease | rs12718244 | A | G | 65642 | 0.0761709 | 0.0100437 | 0.000875444 | 57.51 |
| 24 | Inflammatory bowel disease | rs12722515 | A | C | 65642 | -0.0989022 | 0.0142955 | 0.000728643 | 47.86 |
| 25 | Inflammatory bowel disease | rs12796489 | A | C | 65642 | -0.760367 | 0.0432241 | 0.004692132 | 309.44 |
| 26 | Inflammatory bowel disease | rs1292053 | G | A | 65642 | 0.0701035 | 0.00982936 | 0.000774303 | 50.86 |
| 27 | Inflammatory bowel disease | rs1297258 | T | C | 65642 | -0.114524 | 0.0100654 | 0.00196831 | 129.45 |
| 28 | Inflammatory bowel disease | rs13107612 | T | C | 65642 | 0.0732561 | 0.0108741 | 0.000690906 | 45.38 |
| 29 | Inflammatory bowel disease | rs13204742 | T | G | 65642 | 0.0916208 | 0.0147604 | 0.000586618 | 38.53 |
| 30 | Inflammatory bowel disease | rs13407913 | G | A | 65642 | 0.0917072 | 0.00988181 | 0.001310336 | 86.12 |
| 31 | Inflammatory bowel disease | rs1363907 | A | G | 65642 | 0.0815026 | 0.0104086 | 0.000933192 | 61.31 |
| 32 | Inflammatory bowel disease | rs140143 | T | G | 65642 | -0.133073 | 0.0114182 | 0.002064929 | 135.82 |
| 33 | Inflammatory bowel disease | rs1420098 | C | T | 65642 | -0.0952951 | 0.0102776 | 0.001307999 | 85.97 |
| 34 | Inflammatory bowel disease | rs1517352 | C | A | 65642 | 0.0778816 | 0.0102944 | 0.000871179 | 57.23 |
| 35 | Inflammatory bowel disease | rs1569328 | T | C | 65642 | -0.0809722 | 0.0136766 | 0.000533706 | 35.05 |
| 36 | Inflammatory bowel disease | rs17293632 | T | C | 65642 | 0.107165 | 0.0116106 | 0.001296139 | 85.19 |
| 37 | Inflammatory bowel disease | rs17651741 | A | G | 65642 | 0.0702476 | 0.0126505 | 0.000469528 | 30.83 |
| 38 | Inflammatory bowel disease | rs17694108 | A | G | 65642 | 0.0857629 | 0.0111165 | 0.000905915 | 59.52 |
| 39 | Inflammatory bowel disease | rs17780256 | C | A | 65642 | -0.083427 | 0.0125693 | 0.000670684 | 44.05 |
| 40 | Inflammatory bowel disease | rs181826 | A | C | 65642 | 0.0820444 | 0.0104471 | 0.000938678 | 61.67 |
| 41 | Inflammatory bowel disease | rs1847472 | A | C | 65642 | -0.0672805 | 0.0108961 | 0.0005805 | 38.13 |
| 42 | Inflammatory bowel disease | rs1990760 | T | C | 65642 | -0.0671151 | 0.0107006 | 0.000598938 | 39.34 |
| 43 | Inflammatory bowel disease | rs2024092 | A | G | 65642 | 0.106813 | 0.0121076 | 0.001184229 | 77.82 |
| 44 | Inflammatory bowel disease | rs2050392 | A | G | 65642 | 0.0691178 | 0.0102921 | 0.000686581 | 45.10 |
| 45 | Inflammatory bowel disease | rs2143178 | C | T | 65642 | -0.176684 | 0.0137017 | 0.002526766 | 166.28 |
| 46 | Inflammatory bowel disease | rs2153283 | A | C | 65642 | -0.0859637 | 0.0127458 | 0.00069249 | 45.49 |
| 47 | Inflammatory bowel disease | rs2270395 | T | C | 65642 | 0.0778411 | 0.0118554 | 0.000656324 | 43.11 |
| 48 | Inflammatory bowel disease | rs2274351 | T | C | 65642 | 0.0604993 | 0.0104442 | 0.000510913 | 33.55 |
| 49 | Inflammatory bowel disease | rs2297559 | A | G | 65642 | 0.0741659 | 0.0110454 | 0.000686382 | 45.09 |
| 50 | Inflammatory bowel disease | rs2328546 | C | T | 65642 | 0.0940162 | 0.0126938 | 0.000834983 | 54.85 |
| 51 | Inflammatory bowel disease | rs2395022 | C | A | 65642 | -0.181635 | 0.0233963 | 0.000917327 | 60.27 |
| 52 | Inflammatory bowel disease | rs2488397 | C | G | 65642 | 0.09881 | 0.0121644 | 0.001004159 | 65.98 |
| 53 | Inflammatory bowel disease | rs2497318 | T | C | 65642 | -0.0635464 | 0.00989735 | 0.00062761 | 41.22 |
| 54 | Inflammatory bowel disease | rs2538470 | G | A | 65642 | -0.0675599 | 0.0101646 | 0.000672548 | 44.18 |
| 55 | Inflammatory bowel disease | rs259964 | G | A | 65642 | -0.0674584 | 0.00983489 | 0.00071621 | 47.05 |
| 56 | Inflammatory bowel disease | rs2688608 | T | G | 65642 | 0.062403 | 0.00988642 | 0.000606579 | 39.84 |
| 57 | Inflammatory bowel disease | rs272882 | T | G | 65642 | 0.166117 | 0.0108875 | 0.003533886 | 232.79 |
| 58 | Inflammatory bowel disease | rs2836883 | A | G | 65642 | -0.168413 | 0.0115451 | 0.003231234 | 212.79 |
| 59 | Inflammatory bowel disease | rs2847278 | T | C | 65642 | -0.144528 | 0.0132236 | 0.001816491 | 119.45 |
| 60 | Inflammatory bowel disease | rs2974935 | T | G | 65642 | 0.0687256 | 0.0100715 | 0.000708858 | 46.56 |
| 61 | Inflammatory bowel disease | rs3024493 | A | C | 65642 | 0.196922 | 0.0131753 | 0.003391643 | 223.39 |
| 62 | Inflammatory bowel disease | rs34779708 | G | T | 65642 | 0.106679 | 0.0102408 | 0.001650407 | 108.51 |
| 63 | Inflammatory bowel disease | rs34804116 | A | C | 65642 | -0.0574631 | 0.0104322 | 0.000462002 | 30.34 |
| 64 | Inflammatory bowel disease | rs34856868 | A | G | 65642 | -0.195275 | 0.0340546 | 0.000500659 | 32.88 |
| 65 | Inflammatory bowel disease | rs35164067 | A | G | 65642 | -0.11754 | 0.0127318 | 0.001296721 | 85.23 |
| 66 | Inflammatory bowel disease | rs35256947 | C | T | 65642 | 0.0821647 | 0.0113176 | 0.00080229 | 52.70 |
| 67 | Inflammatory bowel disease | rs35730213 | C | G | 65642 | -0.159624 | 0.0113656 | 0.002995896 | 197.24 |
| 68 | Inflammatory bowel disease | rs36048684 | A | T | 65642 | -0.0941465 | 0.0159649 | 0.000529498 | 34.77 |
| 69 | Inflammatory bowel disease | rs367569 | T | C | 65642 | -0.0958166 | 0.011275 | 0.001098978 | 72.22 |
| 70 | Inflammatory bowel disease | rs3776414 | G | T | 65642 | 0.0773716 | 0.0101613 | 0.000882469 | 57.98 |
| 71 | Inflammatory bowel disease | rs3801835 | T | C | 65642 | 0.064107 | 0.0106005 | 0.000556845 | 36.57 |
| 72 | Inflammatory bowel disease | rs3853824 | C | T | 65642 | 0.064031 | 0.0104099 | 0.000576044 | 37.83 |
| 73 | Inflammatory bowel disease | rs4692386 | C | T | 65642 | 0.0579752 | 0.0101746 | 0.000494371 | 32.47 |
| 74 | Inflammatory bowel disease | rs4703855 | T | C | 65642 | -0.0710611 | 0.0109034 | 0.000646662 | 42.47 |
| 75 | Inflammatory bowel disease | rs4743820 | T | C | 65642 | 0.0639523 | 0.0108526 | 0.000528729 | 34.72 |
| 76 | Inflammatory bowel disease | rs4795397 | G | A | 65642 | 0.138343 | 0.00996661 | 0.002926609 | 192.67 |
| 77 | Inflammatory bowel disease | rs4976646 | C | T | 65642 | 0.0730113 | 0.0104794 | 0.000738932 | 48.54 |
| 78 | Inflammatory bowel disease | rs516246 | T | C | 65642 | 0.0755599 | 0.0101797 | 0.000838623 | 55.09 |
| 79 | Inflammatory bowel disease | rs55808324 | A | G | 65642 | 0.141213 | 0.016844 | 0.001069578 | 70.28 |
| 80 | Inflammatory bowel disease | rs559928 | C | T | 65642 | 0.094388 | 0.0129645 | 0.000806844 | 53.00 |
| 81 | Inflammatory bowel disease | rs56167332 | A | C | 65642 | 0.155855 | 0.0104967 | 0.003347325 | 220.46 |
| 82 | Inflammatory bowel disease | rs6058869 | T | C | 65642 | 0.0556615 | 0.0100037 | 0.000471413 | 30.96 |
| 83 | Inflammatory bowel disease | rs6062496 | A | G | 65642 | 0.123216 | 0.0102312 | 0.002204655 | 145.03 |
| 84 | Inflammatory bowel disease | rs6074022 | T | C | 65642 | -0.0742587 | 0.0114338 | 0.000642174 | 42.18 |
| 85 | Inflammatory bowel disease | rs6111031 | T | C | 65642 | -0.264091 | 0.0147557 | 0.004856145 | 320.31 |
| 86 | Inflammatory bowel disease | rs62037363 | C | T | 65642 | 0.0987549 | 0.0102618 | 0.001408887 | 92.61 |
| 87 | Inflammatory bowel disease | rs62434177 | A | G | 65642 | -0.179105 | 0.031375 | 0.000496193 | 32.59 |
| 88 | Inflammatory bowel disease | rs6456426 | A | C | 65642 | -0.0643405 | 0.0099028 | 0.000642676 | 42.21 |
| 89 | Inflammatory bowel disease | rs6466198 | T | A | 65642 | 0.0841312 | 0.0102451 | 0.001026252 | 67.43 |
| 90 | Inflammatory bowel disease | rs648541 | G | A | 65642 | -0.0648616 | 0.0106716 | 0.000562458 | 36.94 |
| 91 | Inflammatory bowel disease | rs6500315 | G | A | 65642 | 0.0766082 | 0.0118778 | 0.000633318 | 41.60 |
| 92 | Inflammatory bowel disease | rs6561151 | A | G | 65642 | 0.1 | 0.0118661 | 0.001080768 | 71.02 |
| 93 | Inflammatory bowel disease | rs6584281 | G | A | 65642 | -0.164639 | 0.00992861 | 0.004171491 | 274.96 |
| 94 | Inflammatory bowel disease | rs6588248 | G | T | 65642 | 0.0819867 | 0.0099185 | 0.001039827 | 68.33 |
| 95 | Inflammatory bowel disease | rs6651252 | C | T | 65642 | -0.0908484 | 0.014833 | 0.000571145 | 37.51 |
| 96 | Inflammatory bowel disease | rs6708373 | G | A | 65642 | 0.134178 | 0.0099341 | 0.002771523 | 182.43 |
| 97 | Inflammatory bowel disease | rs6740462 | A | C | 65642 | 0.0799597 | 0.0116057 | 0.00072261 | 47.47 |
| 98 | Inflammatory bowel disease | rs6745185 | G | T | 65642 | 0.0698267 | 0.0115253 | 0.000558874 | 36.71 |
| 99 | Inflammatory bowel disease | rs67643815 | T | G | 65642 | -0.0629092 | 0.0101798 | 0.000581454 | 38.19 |
| 100 | Inflammatory bowel disease | rs6933404 | C | T | 65642 | 0.0957518 | 0.0122643 | 0.000927734 | 60.95 |
| 101 | Inflammatory bowel disease | rs7011507 | A | G | 65642 | -0.0846011 | 0.0150825 | 0.000479088 | 31.46 |
| 102 | Inflammatory bowel disease | rs7015630 | C | T | 65642 | -0.0627799 | 0.0113167 | 0.000468615 | 30.77 |
| 103 | Inflammatory bowel disease | rs71593329 | G | T | 65642 | -0.0978042 | 0.0126731 | 0.000906512 | 59.56 |
| 104 | Inflammatory bowel disease | rs7194886 | T | C | 65642 | -0.126026 | 0.0100135 | 0.002407243 | 158.39 |
| 105 | Inflammatory bowel disease | rs7240004 | G | A | 65642 | -0.0665215 | 0.0102898 | 0.000636285 | 41.79 |
| 106 | Inflammatory bowel disease | rs7253253 | T | G | 65642 | -0.134424 | 0.02313 | 0.000514277 | 33.77 |
| 107 | Inflammatory bowel disease | rs72634258 | C | T | 65642 | -0.126877 | 0.0139965 | 0.001250264 | 82.17 |
| 108 | Inflammatory bowel disease | rs72924296 | G | A | 65642 | -0.0638392 | 0.0112629 | 0.000489193 | 32.13 |
| 109 | Inflammatory bowel disease | rs744166 | G | A | 65642 | -0.100017 | 0.0102076 | 0.00146044 | 96.00 |
| 110 | Inflammatory bowel disease | rs7523442 | T | C | 65642 | 0.124537 | 0.00990061 | 0.002404615 | 158.22 |
| 111 | Inflammatory bowel disease | rs7547569 | C | T | 65642 | -0.6472 | 0.0232516 | 0.011665238 | 774.74 |
| 112 | Inflammatory bowel disease | rs7608910 | G | A | 65642 | 0.126444 | 0.0100484 | 0.002406437 | 158.34 |
| 113 | Inflammatory bowel disease | rs7657746 | G | A | 65642 | -0.0868542 | 0.0118004 | 0.000824609 | 54.17 |
| 114 | Inflammatory bowel disease | rs769177 | T | C | 65642 | 0.260903 | 0.0285603 | 0.001269694 | 83.45 |
| 115 | Inflammatory bowel disease | rs7711427 | C | A | 65642 | 0.174764 | 0.0101797 | 0.00446999 | 294.73 |
| 116 | Inflammatory bowel disease | rs7773324 | A | G | 65642 | 0.061818 | 0.0106192 | 0.000515989 | 33.89 |
| 117 | Inflammatory bowel disease | rs780094 | C | T | 65642 | -0.0783055 | 0.00996426 | 0.000939949 | 61.76 |
| 118 | Inflammatory bowel disease | rs7848647 | C | T | 65642 | 0.13239 | 0.0106897 | 0.002331223 | 153.38 |
| 119 | Inflammatory bowel disease | rs78487399 | C | G | 65642 | -0.132093 | 0.0163913 | 0.000988375 | 64.94 |
| 120 | Inflammatory bowel disease | rs79980175 | C | A | 65642 | -0.0953162 | 0.0148313 | 0.000628811 | 41.30 |
| 121 | Inflammatory bowel disease | rs8127691 | C | T | 65642 | -0.114259 | 0.0100818 | 0.001952873 | 128.44 |
| 122 | Inflammatory bowel disease | rs913678 | C | T | 65642 | -0.0691643 | 0.0105418 | 0.000655342 | 43.04 |
| 123 | Inflammatory bowel disease | rs9264942 | C | T | 65642 | 0.094692 | 0.0107773 | 0.001174666 | 77.20 |
| 124 | Inflammatory bowel disease | rs9273363 | A | C | 65642 | -0.193129 | 0.0120075 | 0.003925543 | 258.69 |
| 125 | Inflammatory bowel disease | rs941823 | C | T | 65642 | 0.0830172 | 0.0115361 | 0.000788304 | 51.79 |
| 126 | Inflammatory bowel disease | rs9457247 | T | C | 65642 | 0.089151 | 0.0102086 | 0.00116047 | 76.26 |
| 127 | Inflammatory bowel disease | rs9557207 | G | A | 65642 | -0.0878448 | 0.0120782 | 0.000805186 | 52.89 |
| 128 | Inflammatory bowel disease | rs974801 | G | A | 65642 | -0.0727718 | 0.0101382 | 0.0007843 | 51.52 |
| 129 | Inflammatory bowel disease | rs9836291 | A | G | 65642 | 0.160867 | 0.0105244 | 0.003546618 | 233.63 |
| 130 | Inflammatory bowel disease | rs9889296 | A | G | 65642 | -0.104999 | 0.0112854 | 0.001316989 | 86.56 |
| 1 | Multiple sclerosis | rs1014486 | C | T | 38589 | 0.10075 | 0.0165979 | 0.000953907 | 36.84 |
| 2 | Multiple sclerosis | rs1021156 | C | T | 38589 | -0.115113 | 0.0186355 | 0.000987811 | 38.15 |
| 3 | Multiple sclerosis | rs10420809 | T | C | 38589 | 0.121332 | 0.0210163 | 0.000862978 | 33.33 |
| 4 | Multiple sclerosis | rs1077667 | T | C | 38589 | -0.151862 | 0.0208049 | 0.001378809 | 53.28 |
| 5 | Multiple sclerosis | rs11154801 | A | C | 38589 | 0.102557 | 0.0170438 | 0.000937404 | 36.21 |
| 6 | Multiple sclerosis | rs1131265 | C | G | 38589 | 0.173953 | 0.0220662 | 0.001607853 | 62.14 |
| 7 | Multiple sclerosis | rs115266049 | A | G | 38589 | 0.387301 | 0.0649399 | 0.000920896 | 35.57 |
| 8 | Multiple sclerosis | rs11554159 | A | G | 38589 | -0.1415 | 0.0192663 | 0.001395874 | 53.94 |
| 9 | Multiple sclerosis | rs115985474 | T | C | 38589 | -0.251537 | 0.0459673 | 0.000775364 | 29.94 |
| 10 | Multiple sclerosis | rs11865086 | A | C | 38589 | -0.0934903 | 0.0164939 | 0.000831881 | 32.13 |
| 11 | Multiple sclerosis | rs12087340 | T | C | 38589 | 0.19721 | 0.0286886 | 0.00122305 | 47.25 |
| 12 | Multiple sclerosis | rs12210359 | T | C | 38589 | -0.381855 | 0.0377394 | 0.002646018 | 102.37 |
| 13 | Multiple sclerosis | rs12927355 | T | C | 38589 | -0.193097 | 0.018079 | 0.00294752 | 114.07 |
| 14 | Multiple sclerosis | rs1359062 | G | C | 38589 | -0.162119 | 0.0221213 | 0.001389885 | 53.71 |
| 15 | Multiple sclerosis | rs17066096 | G | A | 38589 | 0.131028 | 0.0190212 | 0.001228161 | 47.45 |
| 16 | Multiple sclerosis | rs1813375 | T | G | 38589 | 0.143234 | 0.0165781 | 0.001930723 | 74.64 |
| 17 | Multiple sclerosis | rs2104286 | C | T | 38589 | -0.189794 | 0.0194055 | 0.002472729 | 95.65 |
| 18 | Multiple sclerosis | rs212405 | T | A | 38589 | 0.139762 | 0.017738 | 0.001606228 | 62.08 |
| 19 | Multiple sclerosis | rs2857700 | C | T | 38589 | -0.681075 | 0.0223316 | 0.023536543 | 930.10 |
| 20 | Multiple sclerosis | rs3129727 | T | C | 38589 | -0.640801 | 0.0622141 | 0.002741655 | 106.08 |
| 21 | Multiple sclerosis | rs34383631 | T | C | 38589 | 0.105261 | 0.0167604 | 0.001021078 | 39.44 |
| 22 | Multiple sclerosis | rs3748817 | C | T | 38589 | -0.129272 | 0.0181572 | 0.00131183 | 50.69 |
| 23 | Multiple sclerosis | rs41286801 | T | C | 38589 | 0.181488 | 0.0225487 | 0.001675949 | 64.78 |
| 24 | Multiple sclerosis | rs4410871 | C | T | 38589 | 0.112435 | 0.0187022 | 0.000935724 | 36.14 |
| 25 | Multiple sclerosis | rs4780355 | C | T | 38589 | -0.10075 | 0.0182211 | 0.00079165 | 30.57 |
| 26 | Multiple sclerosis | rs4796791 | C | T | 38589 | -0.0962189 | 0.0171367 | 0.000816297 | 31.52 |
| 27 | Multiple sclerosis | rs4944958 | G | A | 38589 | -0.107059 | 0.0185652 | 0.000861013 | 33.25 |
| 28 | Multiple sclerosis | rs4976646 | C | T | 38589 | 0.123102 | 0.0171486 | 0.001333613 | 51.53 |
| 29 | Multiple sclerosis | rs60600003 | G | T | 38589 | 0.149282 | 0.0268082 | 0.000802911 | 31.01 |
| 30 | Multiple sclerosis | rs6677309 | C | A | 38589 | -0.29267 | 0.0264562 | 0.003161279 | 122.37 |
| 31 | Multiple sclerosis | rs67297943 | C | T | 38589 | -0.111541 | 0.0205287 | 0.000764453 | 29.52 |
| 32 | Multiple sclerosis | rs6881706 | T | G | 38589 | -0.110647 | 0.0189559 | 0.000882152 | 34.07 |
| 33 | Multiple sclerosis | rs706015 | G | T | 38589 | 0.129272 | 0.0212989 | 0.000953711 | 36.84 |
| 34 | Multiple sclerosis | rs71624119 | A | G | 38589 | -0.116894 | 0.019729 | 0.000908899 | 35.10 |
| 35 | Multiple sclerosis | rs74796499 | A | C | 38589 | -0.27079 | 0.041786 | 0.001087096 | 41.99 |
| 36 | Multiple sclerosis | rs7783 | G | A | 38589 | 0.0989399 | 0.0171056 | 0.000866216 | 33.45 |
| 37 | Multiple sclerosis | rs7923837 | A | G | 38589 | -0.10075 | 0.0170747 | 0.000901423 | 34.81 |
| 38 | Multiple sclerosis | rs8070345 | C | T | 38589 | -0.134531 | 0.016723 | 0.001674268 | 64.71 |
| 39 | Multiple sclerosis | rs842639 | A | G | 38589 | 0.107957 | 0.0180902 | 0.000922042 | 35.61 |
| 40 | Multiple sclerosis | rs9277535 | G | A | 38589 | 0.127513 | 0.0190882 | 0.001155084 | 44.62 |
| 41 | Multiple sclerosis | rs9282641 | A | G | 38589 | -0.193921 | 0.0304428 | 0.001050414 | 40.57 |
| 42 | Multiple sclerosis | rs9736016 | A | T | 38589 | -0.0971267 | 0.0173606 | 0.000810461 | 31.30 |
| 43 | Multiple sclerosis | rs9967792 | C | T | 38589 | 0.102557 | 0.0170285 | 0.000939088 | 36.27 |
| 44 | Multiple sclerosis | rs9989735 | C | G | 38589 | 0.156149 | 0.0209432 | 0.001438479 | 55.59 |
| 1 | Primary biliary cholangitis | rs10488631 | C | T | 13239 | 0.459827 | 0.0465409 | 0.007319371 | 97.60 |
| 2 | Primary biliary cholangitis | rs12924729 | A | G | 13239 | -0.271809 | 0.0356332 | 0.004375808 | 58.18 |
| 3 | Primary biliary cholangitis | rs1372072 | A | G | 13239 | 0.179985 | 0.0327758 | 0.002272599 | 30.15 |
| 4 | Primary biliary cholangitis | rs17641524 | T | C | 13239 | 0.255185 | 0.0375019 | 0.003485237 | 46.30 |
| 5 | Primary biliary cholangitis | rs2069235 | A | G | 13239 | 0.237362 | 0.035481 | 0.003369069 | 44.75 |
| 6 | Primary biliary cholangitis | rs2293370 | A | G | 13239 | -0.34814 | 0.0443656 | 0.004629613 | 61.57 |
| 7 | Primary biliary cholangitis | rs2297067 | T | C | 13239 | 0.324038 | 0.0364664 | 0.005928822 | 78.95 |
| 8 | Primary biliary cholangitis | rs2304256 | A | C | 13239 | -0.241435 | 0.0373758 | 0.003141938 | 41.72 |
| 9 | Primary biliary cholangitis | rs35464393 | C | T | 13239 | 0.388018 | 0.0534412 | 0.00396616 | 52.71 |
| 10 | Primary biliary cholangitis | rs3745516 | G | A | 13239 | -0.332392 | 0.0356822 | 0.006511865 | 86.76 |
| 11 | Primary biliary cholangitis | rs3771317 | C | T | 13239 | 0.344157 | 0.0450977 | 0.004379686 | 58.23 |
| 12 | Primary biliary cholangitis | rs4780355 | C | T | 13239 | -0.245134 | 0.0364432 | 0.003405942 | 45.24 |
| 13 | Primary biliary cholangitis | rs485499 | C | T | 13239 | -0.345311 | 0.034509 | 0.007506347 | 100.11 |
| 14 | Primary biliary cholangitis | rs4938573 | T | C | 13239 | 0.332261 | 0.0447638 | 0.004144251 | 55.09 |
| 15 | Primary biliary cholangitis | rs510372 | T | C | 13239 | -0.205672 | 0.034132 | 0.002735157 | 36.30 |
| 16 | Primary biliary cholangitis | rs6679356 | T | C | 13239 | -0.416998 | 0.0381197 | 0.008957894 | 119.65 |
| 17 | Primary biliary cholangitis | rs7665090 | G | A | 13239 | 0.198939 | 0.0323963 | 0.002840263 | 37.70 |
| 18 | Primary biliary cholangitis | rs7774434 | C | T | 13239 | 0.51802 | 0.0327504 | 0.018547021 | 250.15 |
| 19 | Primary biliary cholangitis | rs860413 | C | A | 13239 | -0.247949 | 0.0376861 | 0.003259038 | 43.28 |
| 20 | Primary biliary cholangitis | rs911263 | T | C | 13239 | 0.218529 | 0.0365505 | 0.002692807 | 35.74 |
| 21 | Primary biliary cholangitis | rs928976 | T | C | 13239 | 0.422912 | 0.0341849 | 0.011428371 | 153.03 |
| 22 | Primary biliary cholangitis | rs9303277 | T | C | 13239 | 0.215434 | 0.0323021 | 0.003348537 | 44.47 |
| 23 | Primary biliary cholangitis | rs9591325 | C | T | 13239 | -0.48988 | 0.0758756 | 0.003138735 | 41.68 |
| 1 | Psoriasis | rs113866081 | A | G | 33394 | -1.72766 | 0.228118 | 0.001714682 | 57.36 |
| 2 | Psoriasis | rs114071237 | T | G | 33394 | -1.32539 | 0.199955 | 0.001313964 | 43.93 |
| 3 | Psoriasis | rs114835245 | G | A | 33394 | -0.986981 | 0.14747 | 0.001339552 | 44.79 |
| 4 | Psoriasis | rs116054851 | C | A | 33394 | -3.77226 | 0.433175 | 0.002265807 | 75.83 |
| 5 | Psoriasis | rs11648503 | A | G | 33394 | 0.264617 | 0.0430029 | 0.001132607 | 37.86 |
| 6 | Psoriasis | rs11652075 | T | C | 33394 | -0.169743 | 0.0303503 | 0.000935799 | 31.28 |
| 7 | Psoriasis | rs116976310 | C | A | 33394 | -1.50418 | 0.256406 | 0.001029503 | 34.41 |
| 8 | Psoriasis | rs117094752 | C | T | 33394 | -0.785701 | 0.122014 | 0.001240189 | 41.46 |
| 9 | Psoriasis | rs117135073 | T | C | 33394 | -2.42362 | 0.345187 | 0.001474046 | 49.29 |
| 10 | Psoriasis | rs117505735 | A | C | 33394 | -2.12695 | 0.384995 | 0.000913144 | 30.52 |
| 11 | Psoriasis | rs117950837 | C | A | 33394 | -1.17118 | 0.212714 | 0.000906969 | 30.31 |
| 12 | Psoriasis | rs12135210 | T | G | 33394 | -1.09154 | 0.189249 | 0.000995202 | 33.26 |
| 13 | Psoriasis | rs13394291 | C | T | 33394 | -1.751 | 0.214216 | 0.001996788 | 66.81 |
| 14 | Psoriasis | rs141417497 | A | G | 33394 | -3.48676 | 0.503652 | 0.00143315 | 47.92 |
| 15 | Psoriasis | rs146281313 | C | A | 33394 | -4.60517 | 0.400357 | 0.003946487 | 132.30 |
| 16 | Psoriasis | rs17066690 | C | A | 33394 | -2.68972 | 0.351247 | 0.001752906 | 58.64 |
| 17 | Psoriasis | rs17728338 | A | G | 33394 | 0.448972 | 0.0577294 | 0.001807965 | 60.48 |
| 18 | Psoriasis | rs1990760 | T | C | 33394 | 0.192251 | 0.0342314 | 0.000943647 | 31.54 |
| 19 | Psoriasis | rs2229092 | C | A | 33394 | -0.42986 | 0.0749622 | 0.000983725 | 32.88 |
| 20 | Psoriasis | rs2277268 | A | G | 33394 | -0.472567 | 0.0848388 | 0.000928252 | 31.02 |
| 21 | Psoriasis | rs2329570 | C | T | 33394 | -4.29769 | 0.577387 | 0.001656334 | 55.40 |
| 22 | Psoriasis | rs2523554 | T | C | 33394 | 0.192251 | 0.0326113 | 0.001039635 | 34.75 |
| 23 | Psoriasis | rs30376 | T | C | 33394 | -0.209207 | 0.0336584 | 0.001155566 | 38.63 |
| 24 | Psoriasis | rs3135952 | T | C | 33394 | -1.92552 | 0.227343 | 0.002143545 | 71.73 |
| 25 | Psoriasis | rs34413922 | T | C | 33394 | -1.9119 | 0.216556 | 0.002328675 | 77.94 |
| 26 | Psoriasis | rs4406273 | A | G | 33394 | 1.44478 | 0.0415524 | 0.034937996 | 1,208.89 |
| 27 | Psoriasis | rs4845454 | T | C | 33394 | 0.268533 | 0.0338106 | 0.001885391 | 63.08 |
| 28 | Psoriasis | rs57939339 | A | G | 33394 | -1.49032 | 0.223364 | 0.00133133 | 44.52 |
| 29 | Psoriasis | rs6031647 | G | A | 33394 | 0.388455 | 0.0624968 | 0.001155567 | 38.63 |
| 30 | Psoriasis | rs61774731 | A | G | 33394 | -2.63387 | 0.288229 | 0.002494366 | 83.50 |
| 31 | Psoriasis | rs6714339 | T | C | 33394 | -0.37295 | 0.058678 | 0.001208251 | 40.39 |
| 32 | Psoriasis | rs67543742 | G | A | 33394 | 0.211961 | 0.0369674 | 0.000983509 | 32.87 |
| 33 | Psoriasis | rs6755395 | G | A | 33394 | -1.79999 | 0.295287 | 0.001111476 | 37.16 |
| 34 | Psoriasis | rs6833586 | G | A | 33394 | -4.16048 | 0.556202 | 0.001672732 | 55.95 |
| 35 | Psoriasis | rs72669160 | C | T | 33394 | -0.450515 | 0.0659883 | 0.001393832 | 46.61 |
| 36 | Psoriasis | rs73277117 | C | A | 33394 | -3.65351 | 0.492714 | 0.001643796 | 54.98 |
| 37 | Psoriasis | rs73695700 | A | G | 33394 | 0.889166 | 0.112882 | 0.001854563 | 62.04 |
| 38 | Psoriasis | rs74864202 | A | C | 33394 | -3.61192 | 0.461446 | 0.001831344 | 61.26 |
| 39 | Psoriasis | rs75430970 | C | T | 33394 | -0.284487 | 0.0473042 | 0.001081899 | 36.17 |
| 40 | Psoriasis | rs76930577 | C | T | 33394 | -3.64966 | 0.3803 | 0.002750348 | 92.09 |
| 41 | Psoriasis | rs77464075 | T | C | 33394 | -1.79276 | 0.300635 | 0.001063738 | 35.56 |
| 42 | Psoriasis | rs77520588 | A | G | 33394 | -1.63015 | 0.208855 | 0.001820981 | 60.92 |
| 43 | Psoriasis | rs77840275 | C | T | 33394 | -0.438195 | 0.0695854 | 0.001186082 | 39.65 |
| 44 | Psoriasis | rs78580783 | C | A | 33394 | -1.36297 | 0.20981 | 0.001262128 | 42.20 |
| 45 | Psoriasis | rs78636848 | A | C | 33394 | -1.6513 | 0.244989 | 0.001358626 | 45.43 |
| 46 | Psoriasis | rs7865117 | T | G | 33394 | -2.73337 | 0.421661 | 0.001256769 | 42.02 |
| 47 | Psoriasis | rs80174646 | T | G | 33394 | -0.445975 | 0.0760664 | 0.0010283 | 34.37 |
| 48 | Psoriasis | rs892085 | A | G | 33394 | 0.182362 | 0.0291918 | 0.00116727 | 39.02 |
| 49 | Psoriasis | rs9525864 | G | A | 33394 | -0.552169 | 0.0628269 | 0.002307709 | 77.24 |
| 1 | Rheumatoid arthritis | rs10435844 | T | G | 58284 | -0.0784 | 0.0121 | 0.000719779 | 41.98 |
| 2 | Rheumatoid arthritis | rs10911902 | T | C | 58284 | -0.0847 | 0.0152 | 0.000532475 | 31.05 |
| 3 | Rheumatoid arthritis | rs11123811 | C | T | 58284 | -0.0995 | 0.0114 | 0.001305329 | 76.18 |
| 4 | Rheumatoid arthritis | rs112733823 | T | C | 58284 | 0.191 | 0.0188 | 0.001767801 | 103.21 |
| 5 | Rheumatoid arthritis | rs114508013 | A | G | 58284 | 0.488 | 0.0441 | 0.002096533 | 122.45 |
| 6 | Rheumatoid arthritis | rs115521560 | C | A | 58284 | 0.7878 | 0.0364 | 0.007972664 | 468.40 |
| 7 | Rheumatoid arthritis | rs11574914 | A | G | 58284 | 0.1153 | 0.0149 | 0.001026339 | 59.88 |
| 8 | Rheumatoid arthritis | rs117026326 | T | C | 58284 | 0.381 | 0.0424 | 0.001383463 | 80.74 |
| 9 | Rheumatoid arthritis | rs11754264 | C | T | 58284 | -0.1359 | 0.0193 | 0.000849974 | 49.58 |
| 10 | Rheumatoid arthritis | rs11889341 | T | C | 58284 | 0.1466 | 0.0129 | 0.002210945 | 129.14 |
| 11 | Rheumatoid arthritis | rs12126142 | A | G | 58284 | -0.0751 | 0.0116 | 0.000718625 | 41.91 |
| 12 | Rheumatoid arthritis | rs1234313 | G | A | 58284 | 0.0797 | 0.0133 | 0.000615739 | 35.91 |
| 13 | Rheumatoid arthritis | rs12466919 | T | C | 58284 | 0.1025 | 0.0152 | 0.000779601 | 45.47 |
| 14 | Rheumatoid arthritis | rs12530098 | T | C | 58284 | 0.1382 | 0.0204 | 0.0007868 | 45.89 |
| 15 | Rheumatoid arthritis | rs12918327 | T | C | 58284 | 0.0867 | 0.0157 | 0.000522953 | 30.49 |
| 16 | Rheumatoid arthritis | rs13103285 | T | C | 58284 | 0.0989 | 0.0131 | 0.000976959 | 56.99 |
| 17 | Rheumatoid arthritis | rs1355208 | G | A | 58284 | 0.0818 | 0.0119 | 0.000810049 | 47.25 |
| 18 | Rheumatoid arthritis | rs139395255 | G | A | 58284 | 0.3833 | 0.0235 | 0.004543753 | 266.03 |
| 19 | Rheumatoid arthritis | rs146305655 | A | G | 58284 | -0.4379 | 0.0452 | 0.001607773 | 93.86 |
| 20 | Rheumatoid arthritis | rs1538981 | T | C | 58284 | 0.0671 | 0.0114 | 0.000594057 | 34.64 |
| 21 | Rheumatoid arthritis | rs1571878 | T | C | 58284 | -0.1539 | 0.0116 | 0.003010941 | 176.01 |
| 22 | Rheumatoid arthritis | rs1595260 | T | A | 58284 | 0.0845 | 0.0126 | 0.00077106 | 44.97 |
| 23 | Rheumatoid arthritis | rs1611236 | A | G | 58284 | -0.1165 | 0.0131 | 0.001355099 | 79.09 |
| 24 | Rheumatoid arthritis | rs1858037 | A | T | 58284 | -0.1012 | 0.0131 | 0.00102288 | 59.68 |
| 25 | Rheumatoid arthritis | rs1883832 | C | T | 58284 | 0.1052 | 0.0127 | 0.001175882 | 68.61 |
| 26 | Rheumatoid arthritis | rs1893592 | C | A | 58284 | -0.0976 | 0.0132 | 0.00093712 | 54.67 |
| 27 | Rheumatoid arthritis | rs1950897 | T | C | 58284 | 0.1069 | 0.0144 | 0.000944649 | 55.11 |
| 28 | Rheumatoid arthritis | rs2069235 | A | G | 58284 | 0.1296 | 0.014 | 0.001468137 | 85.69 |
| 29 | Rheumatoid arthritis | rs2073609 | C | T | 58284 | 0.1029 | 0.0182 | 0.000548151 | 31.96 |
| 30 | Rheumatoid arthritis | rs2076616 | G | A | 58284 | -0.0885 | 0.0135 | 0.0007368 | 42.97 |
| 31 | Rheumatoid arthritis | rs212389 | A | G | 58284 | 0.1058 | 0.0147 | 0.000887977 | 51.80 |
| 32 | Rheumatoid arthritis | rs2233424 | T | C | 58284 | 0.1964 | 0.0187 | 0.001888988 | 110.30 |
| 33 | Rheumatoid arthritis | rs2258734 | A | G | 58284 | -0.0921 | 0.0123 | 0.000961041 | 56.07 |
| 34 | Rheumatoid arthritis | rs2275806 | A | G | 58284 | -0.0725 | 0.0122 | 0.000605542 | 35.31 |
| 35 | Rheumatoid arthritis | rs2301888 | A | G | 58284 | -0.1282 | 0.0121 | 0.001922296 | 112.25 |
| 36 | Rheumatoid arthritis | rs244685 | G | T | 58284 | -0.089 | 0.0144 | 0.00065497 | 38.20 |
| 37 | Rheumatoid arthritis | rs28411352 | T | C | 58284 | 0.0914 | 0.0136 | 0.000774335 | 45.16 |
| 38 | Rheumatoid arthritis | rs2841275 | C | A | 58284 | 0.1617 | 0.0179 | 0.00139816 | 81.60 |
| 39 | Rheumatoid arthritis | rs28421442 | A | T | 58284 | -0.1234 | 0.0214 | 0.000570172 | 33.25 |
| 40 | Rheumatoid arthritis | rs2847297 | G | A | 58284 | 0.0903 | 0.0119 | 0.000986969 | 57.58 |
| 41 | Rheumatoid arthritis | rs2918392 | C | T | 58284 | 0.0668 | 0.0122 | 0.000514115 | 29.98 |
| 42 | Rheumatoid arthritis | rs3025669 | G | C | 58284 | -0.2534 | 0.0222 | 0.002230428 | 130.28 |
| 43 | Rheumatoid arthritis | rs3087243 | A | G | 58284 | -0.1261 | 0.0124 | 0.0017712 | 103.41 |
| 44 | Rheumatoid arthritis | rs3134883 | A | G | 58284 | 0.0991 | 0.0125 | 0.001077233 | 62.85 |
| 45 | Rheumatoid arthritis | rs34046593 | A | G | 58284 | 0.1422 | 0.017 | 0.001199032 | 69.97 |
| 46 | Rheumatoid arthritis | rs34502849 | A | G | 58284 | -0.0851 | 0.014 | 0.000633546 | 36.95 |
| 47 | Rheumatoid arthritis | rs3757387 | C | T | 58284 | 0.1236 | 0.0137 | 0.00139457 | 81.39 |
| 48 | Rheumatoid arthritis | rs3761959 | T | C | 58284 | 0.0744 | 0.0115 | 0.000717611 | 41.85 |
| 49 | Rheumatoid arthritis | rs3806624 | G | A | 58284 | 0.0863 | 0.0131 | 0.000744057 | 43.40 |
| 50 | Rheumatoid arthritis | rs403214 | G | A | 58284 | -0.0914 | 0.0146 | 0.000671963 | 39.19 |
| 51 | Rheumatoid arthritis | rs42034 | G | A | 58284 | 0.0871 | 0.0153 | 0.000555729 | 32.41 |
| 52 | Rheumatoid arthritis | rs4409785 | C | T | 58284 | 0.0982 | 0.017 | 0.000572173 | 33.37 |
| 53 | Rheumatoid arthritis | rs4602367 | G | A | 58284 | 0.075 | 0.0117 | 0.000704523 | 41.09 |
| 54 | Rheumatoid arthritis | rs4717901 | C | A | 58284 | 0.249 | 0.0349 | 0.000872608 | 50.90 |
| 55 | Rheumatoid arthritis | rs4795400 | T | C | 58284 | 0.0743 | 0.012 | 0.000657325 | 38.34 |
| 56 | Rheumatoid arthritis | rs5020946 | T | G | 58284 | 0.6519 | 0.0169 | 0.024893796 | 1,487.90 |
| 57 | Rheumatoid arthritis | rs502919 | C | T | 58284 | 0.0829 | 0.0134 | 0.000656244 | 38.27 |
| 58 | Rheumatoid arthritis | rs5754104 | A | G | 58284 | 0.0891 | 0.0139 | 0.000704483 | 41.09 |
| 59 | Rheumatoid arthritis | rs6011186 | T | C | 58284 | -0.1074 | 0.0171 | 0.000676353 | 39.45 |
| 60 | Rheumatoid arthritis | rs61828284 | T | C | 58284 | -0.2018 | 0.0348 | 0.000576612 | 33.63 |
| 61 | Rheumatoid arthritis | rs62422878 | T | C | 58284 | 0.1037 | 0.0176 | 0.000595284 | 34.72 |
| 62 | Rheumatoid arthritis | rs6421571 | C | T | 58284 | 0.134 | 0.0178 | 0.0009714 | 56.67 |
| 63 | Rheumatoid arthritis | rs6479800 | C | G | 58284 | 0.1202 | 0.0181 | 0.000756091 | 44.10 |
| 64 | Rheumatoid arthritis | rs660442 | A | G | 58284 | -0.1067 | 0.0175 | 0.000637421 | 37.17 |
| 65 | Rheumatoid arthritis | rs6679677 | A | C | 58284 | 0.591 | 0.023 | 0.01120154 | 660.24 |
| 66 | Rheumatoid arthritis | rs7097397 | A | G | 58284 | -0.0847 | 0.012 | 0.000854051 | 49.82 |
| 67 | Rheumatoid arthritis | rs7105899 | A | G | 58284 | -0.0797 | 0.0128 | 0.00066475 | 38.77 |
| 68 | Rheumatoid arthritis | rs71508903 | T | C | 58284 | 0.1487 | 0.0143 | 0.001851806 | 108.13 |
| 69 | Rheumatoid arthritis | rs71565312 | A | G | 58284 | 0.699 | 0.0402 | 0.005160667 | 302.33 |
| 70 | Rheumatoid arthritis | rs7170107 | T | C | 58284 | 0.1366 | 0.0158 | 0.0012808 | 74.74 |
| 71 | Rheumatoid arthritis | rs7206670 | T | G | 58284 | 0.0701 | 0.0119 | 0.000595024 | 34.70 |
| 72 | Rheumatoid arthritis | rs740122 | A | G | 58284 | -0.0782 | 0.0134 | 0.000583984 | 34.06 |
| 73 | Rheumatoid arthritis | rs76153210 | T | C | 58284 | 0.1597 | 0.0205 | 0.001040162 | 60.69 |
| 74 | Rheumatoid arthritis | rs7731626 | A | G | 58284 | -0.1956 | 0.0184 | 0.001935136 | 113.00 |
| 75 | Rheumatoid arthritis | rs7749323 | A | G | 58284 | 0.2835 | 0.0253 | 0.002149716 | 125.56 |
| 76 | Rheumatoid arthritis | rs8032939 | C | T | 58284 | 0.1244 | 0.0123 | 0.001751942 | 102.29 |
| 77 | Rheumatoid arthritis | rs8126756 | C | T | 58284 | -0.0823 | 0.0137 | 0.000618786 | 36.09 |
| 78 | Rheumatoid arthritis | rs9271365 | G | T | 58284 | 0.4888 | 0.0128 | 0.024409598 | 1,458.24 |
| 79 | Rheumatoid arthritis | rs9405192 | A | G | 58284 | -0.089 | 0.0137 | 0.000723561 | 42.20 |
| 80 | Rheumatoid arthritis | rs9532434 | C | T | 58284 | 0.114 | 0.0126 | 0.001402522 | 81.86 |
| 81 | Rheumatoid arthritis | rs9693589 | A | G | 58284 | 0.1127 | 0.0128 | 0.001328316 | 77.52 |
| 82 | Rheumatoid arthritis | rs9927316 | G | C | 58284 | 0.0906 | 0.0136 | 0.000760849 | 44.38 |
| 83 | Rheumatoid arthritis | rs9943599 | T | C | 58284 | 0.083 | 0.0131 | 0.00068828 | 40.14 |
| 1 | Systemic lupus erythematosus | rs10048743 | T | G | 14267 | -0.231112 | 0.0412056 | 0.002200104 | 31.45 |
| 2 | Systemic lupus erythematosus | rs10200680 | T | C | 14267 | -0.248461 | 0.0424835 | 0.002391681 | 34.20 |
| 3 | Systemic lupus erythematosus | rs1078324 | A | C | 14267 | -0.71335 | 0.0781665 | 0.00580368 | 83.27 |
| 4 | Systemic lupus erythematosus | rs10912578 | G | A | 14267 | -0.24686 | 0.0309918 | 0.004427394 | 63.44 |
| 5 | Systemic lupus erythematosus | rs1143679 | A | G | 14267 | 0.582216 | 0.0399866 | 0.01464201 | 211.97 |
| 6 | Systemic lupus erythematosus | rs12094036 | C | T | 14267 | -0.328504 | 0.0578595 | 0.002254339 | 32.23 |
| 7 | Systemic lupus erythematosus | rs12524498 | T | G | 14267 | -0.673345 | 0.120793 | 0.002173273 | 31.07 |
| 8 | Systemic lupus erythematosus | rs13019891 | T | G | 14267 | -0.562119 | 0.0290336 | 0.025601114 | 374.80 |
| 9 | Systemic lupus erythematosus | rs13136219 | T | C | 14267 | -0.174353 | 0.027787 | 0.002751985 | 39.37 |
| 10 | Systemic lupus erythematosus | rs13332649 | G | A | 14267 | -0.314711 | 0.0375683 | 0.0048946 | 70.16 |
| 11 | Systemic lupus erythematosus | rs143123127 | A | G | 14267 | 0.470004 | 0.0840342 | 0.002187797 | 31.28 |
| 12 | Systemic lupus erythematosus | rs1464446 | T | G | 14267 | -0.328504 | 0.0401497 | 0.004670367 | 66.94 |
| 13 | Systemic lupus erythematosus | rs150180633 | T | C | 14267 | 0.928219 | 0.0689573 | 0.012540848 | 181.17 |
| 14 | Systemic lupus erythematosus | rs17849501 | T | C | 14267 | 0.81093 | 0.0498642 | 0.018200329 | 264.44 |
| 15 | Systemic lupus erythematosus | rs2431697 | C | T | 14267 | -0.223144 | 0.0292964 | 0.004049924 | 58.01 |
| 16 | Systemic lupus erythematosus | rs2459611 | T | C | 14267 | 0.261365 | 0.045245 | 0.002333492 | 33.37 |
| 17 | Systemic lupus erythematosus | rs2573219 | C | A | 14267 | 0.587787 | 0.0429292 | 0.012969766 | 187.44 |
| 18 | Systemic lupus erythematosus | rs268124 | T | C | 14267 | 0.18633 | 0.0323703 | 0.002317032 | 33.13 |
| 19 | Systemic lupus erythematosus | rs34703115 | C | T | 14267 | -0.616186 | 0.104778 | 0.002418239 | 34.58 |
| 20 | Systemic lupus erythematosus | rs35000415 | T | C | 14267 | 0.587787 | 0.041539 | 0.013840205 | 200.20 |
| 21 | Systemic lupus erythematosus | rs35251378 | A | G | 14267 | -0.235722 | 0.0324266 | 0.003690278 | 52.84 |
| 22 | Systemic lupus erythematosus | rs353608 | G | A | 14267 | 0.18633 | 0.0280198 | 0.003090002 | 44.22 |
| 23 | Systemic lupus erythematosus | rs3747093 | A | G | 14267 | 0.262364 | 0.0345055 | 0.004035923 | 57.81 |
| 24 | Systemic lupus erythematosus | rs389884 | G | A | 14267 | 0.928219 | 0.0432319 | 0.031300319 | 460.93 |
| 25 | Systemic lupus erythematosus | rs4274624 | T | C | 14267 | -0.559616 | 0.0326791 | 0.020140544 | 293.21 |
| 26 | Systemic lupus erythematosus | rs4388254 | T | C | 14267 | 0.378436 | 0.0603977 | 0.002744214 | 39.25 |
| 27 | Systemic lupus erythematosus | rs4661543 | G | T | 14267 | 0.274437 | 0.0423755 | 0.002931218 | 41.94 |
| 28 | Systemic lupus erythematosus | rs4916215 | T | C | 14267 | 0.223144 | 0.0339693 | 0.003015456 | 43.15 |
| 29 | Systemic lupus erythematosus | rs58688157 | G | A | 14267 | -0.223144 | 0.0335647 | 0.003088367 | 44.19 |
| 30 | Systemic lupus erythematosus | rs58721818 | T | C | 14267 | 0.65752 | 0.0755941 | 0.00527488 | 75.65 |
| 31 | Systemic lupus erythematosus | rs6671847 | A | G | 14267 | 0.198851 | 0.0289651 | 0.003292612 | 47.12 |
| 32 | Systemic lupus erythematosus | rs6679677 | A | C | 14267 | 0.336472 | 0.0464854 | 0.003658812 | 52.38 |
| 33 | Systemic lupus erythematosus | rs6889239 | C | T | 14267 | 0.277632 | 0.03174 | 0.005334205 | 76.50 |
| 34 | Systemic lupus erythematosus | rs7097397 | A | G | 14267 | -0.18633 | 0.0287118 | 0.002943282 | 42.11 |
| 35 | Systemic lupus erythematosus | rs73068668 | A | G | 14267 | -0.314711 | 0.0574903 | 0.002095998 | 29.96 |
| 36 | Systemic lupus erythematosus | rs7768653 | T | C | 14267 | -0.207014 | 0.0296891 | 0.003396216 | 48.61 |
| 37 | Systemic lupus erythematosus | rs7823055 | T | G | 14267 | -0.350657 | 0.0286208 | 0.010411741 | 150.09 |
| 38 | Systemic lupus erythematosus | rs7899626 | T | C | 14267 | 0.182322 | 0.0332532 | 0.002102638 | 30.06 |
| 39 | Systemic lupus erythematosus | rs9852014 | G | A | 14267 | 0.620577 | 0.0492727 | 0.010996233 | 158.61 |
| 1 | Type 1 diabetes | rs10183097 | C | T | 24840 | 0.2053 | 0.0322 | 0.001633821 | 40.65 |
| 2 | Type 1 diabetes | rs1027769 | T | G | 24840 | -0.9962 | 0.1588 | 0.001581805 | 39.35 |
| 3 | Type 1 diabetes | rs10760335 | G | A | 24840 | 0.1357 | 0.0243 | 0.001253865 | 31.18 |
| 4 | Type 1 diabetes | rs10830227 | A | G | 24840 | 0.1582 | 0.0233 | 0.001852441 | 46.10 |
| 5 | Type 1 diabetes | rs10865468 | C | G | 24840 | -0.1624 | 0.0277 | 0.001381848 | 34.37 |
| 6 | Type 1 diabetes | rs10911399 | G | A | 24840 | -0.3707 | 0.064 | 0.0013488 | 33.55 |
| 7 | Type 1 diabetes | rs11571297 | C | T | 24840 | -0.1964 | 0.0237 | 0.002756993 | 68.67 |
| 8 | Type 1 diabetes | rs12722495 | C | T | 24840 | -0.3145 | 0.0408 | 0.002386337 | 59.41 |
| 9 | Type 1 diabetes | rs13182737 | A | G | 24840 | 0.1465 | 0.0259 | 0.001286368 | 31.99 |
| 10 | Type 1 diabetes | rs17125653 | A | T | 24840 | 0.2355 | 0.0402 | 0.00137968 | 34.32 |
| 11 | Type 1 diabetes | rs17863786 | G | A | 24840 | 0.4144 | 0.0628 | 0.001749879 | 43.54 |
| 12 | Type 1 diabetes | rs185774696 | T | C | 24840 | 0.6489 | 0.0418 | 0.009608559 | 240.97 |
| 13 | Type 1 diabetes | rs1869449 | A | G | 24840 | 0.1769 | 0.0269 | 0.001737977 | 43.24 |
| 14 | Type 1 diabetes | rs192324744 | G | T | 24840 | 0.562 | 0.0875 | 0.001657999 | 41.25 |
| 15 | Type 1 diabetes | rs194749 | C | T | 24840 | -0.1638 | 0.0281 | 0.00136606 | 33.98 |
| 16 | Type 1 diabetes | rs201417739 | C | A | 24840 | -0.416 | 0.0663 | 0.001582414 | 39.37 |
| 17 | Type 1 diabetes | rs202520 | G | A | 24840 | -0.1573 | 0.0256 | 0.001517631 | 37.75 |
| 18 | Type 1 diabetes | rs206763 | A | G | 24840 | 0.6792 | 0.0779 | 0.003050997 | 76.01 |
| 19 | Type 1 diabetes | rs2111485 | G | A | 24840 | 0.1577 | 0.0248 | 0.001625182 | 40.43 |
| 20 | Type 1 diabetes | rs2144013 | G | A | 24840 | 0.2234 | 0.0317 | 0.001995394 | 49.66 |
| 21 | Type 1 diabetes | rs2269247 | T | C | 24840 | 0.1709 | 0.0295 | 0.00134928 | 33.56 |
| 22 | Type 1 diabetes | rs231971 | G | A | 24840 | 0.2411 | 0.0399 | 0.001467774 | 36.51 |
| 23 | Type 1 diabetes | rs34296259 | A | T | 24840 | 0.6637 | 0.1171 | 0.001291567 | 32.12 |
| 24 | Type 1 diabetes | rs34536443 | C | G | 24840 | -0.4139 | 0.0665 | 0.00155711 | 38.74 |
| 25 | Type 1 diabetes | rs4566101 | C | T | 24840 | 0.1755 | 0.0255 | 0.001903246 | 47.36 |
| 26 | Type 1 diabetes | rs506770 | C | G | 24840 | 1.0048 | 0.0426 | 0.021906295 | 556.29 |
| 27 | Type 1 diabetes | rs55996894 | C | G | 24840 | -0.1785 | 0.0323 | 0.001227966 | 30.54 |
| 28 | Type 1 diabetes | rs62410259 | A | G | 24840 | -0.3796 | 0.0533 | 0.002037795 | 50.72 |
| 29 | Type 1 diabetes | rs6679677 | A | C | 24840 | 0.6527 | 0.0346 | 0.014123616 | 355.83 |
| 30 | Type 1 diabetes | rs6719660 | G | A | 24840 | 0.2918 | 0.0524 | 0.001246851 | 31.01 |
| 31 | Type 1 diabetes | rs689 | T | A | 24840 | 0.7004 | 0.0354 | 0.0155147 | 391.43 |
| 32 | Type 1 diabetes | rs6909461 | C | A | 24840 | -0.314 | 0.0332 | 0.003588149 | 89.44 |
| 33 | Type 1 diabetes | rs741172 | T | C | 24840 | -0.2034 | 0.0258 | 0.002495891 | 62.15 |
| 34 | Type 1 diabetes | rs77523242 | C | T | 24840 | -0.3705 | 0.0635 | 0.001368619 | 34.04 |
| 35 | Type 1 diabetes | rs79075295 | A | G | 24840 | -0.4192 | 0.0621 | 0.001831099 | 45.56 |
| 36 | Type 1 diabetes | rs8056814 | A | G | 24840 | 0.2641 | 0.0415 | 0.001627727 | 40.50 |
| 37 | Type 1 diabetes | rs9273363 | A | C | 24840 | 1.2786 | 0.0334 | 0.05570965 | 1,465.35 |
| 38 | Type 1 diabetes | rs9296062 | C | G | 24840 | 0.6913 | 0.054 | 0.006554477 | 163.87 |
| 1 | Myasthenia gravis | rs2245569 | G | A | 38243 | 0.2369 | 0.042 | 0.000831225 | 31.81 |
| 2 | Myasthenia gravis | rs35274388 | A | G | 38243 | 0.4495 | 0.0812 | 0.000800659 | 30.64 |
| 3 | Myasthenia gravis | rs4409785 | C | T | 38243 | 0.2545 | 0.045 | 0.000835671 | 31.98 |
| 4 | Myasthenia gravis | rs4574025 | T | C | 38243 | -0.2874 | 0.0369 | 0.001583728 | 60.66 |
| 5 | Myasthenia gravis | rs6679677 | A | C | 38243 | 0.3937 | 0.0613 | 0.001077432 | 41.25 |
| 6 | Myasthenia gravis | rs76815088 | C | T | 38243 | -0.8661 | 0.1128 | 0.001539207 | 58.95 |

**Table S2** Details of all SNPs for breast cancer

|  | **Exposure** | **SNP** | **Effect allele** | **Other allele** | **Sample size** | **Beta** | **SE** | **R2** | **F** |
| --- | --- | --- | --- | --- | --- | --- | --- | --- | --- |
| 1 | Breast cancer | rs1910020 | C | T | 32498 | -0.1461 | 0.0193 | 0.001760208 | 57.30 |
| 2 | Breast cancer | rs9933302 | C | T | 32498 | 0.1303 | 0.0215 | 0.001128924 | 36.73 |
| 3 | Breast cancer | rs3095602 | A | G | 32498 | 0.2281 | 0.0197 | 0.004108408 | 134.06 |
| 4 | Breast cancer | rs487930 | A | C | 32498 | 0.1227 | 0.0175 | 0.001510428 | 49.16 |
| 5 | Breast cancer | rs548980 | T | C | 32498 | 0.0984 | 0.018 | 0.000918733 | 29.88 |
| 6 | Breast cancer | rs6721996 | A | G | 32498 | -0.1371 | 0.0173 | 0.001928802 | 62.80 |
| 7 | Breast cancer | rs719338 | T | G | 32498 | -0.107 | 0.0178 | 0.001110678 | 36.13 |
| 8 | Breast cancer | rs9371545 | A | G | 32498 | 0.2775 | 0.0339 | 0.002057668 | 67.00 |
| 9 | Breast cancer | rs9693995 | T | C | 32498 | -0.1244 | 0.0176 | 0.001534941 | 49.96 |
| 10 | Breast cancer | rs10483813 | A | T | 32498 | -0.1427 | 0.0208 | 0.001446226 | 47.06 |
| 11 | Breast cancer | rs132289 | G | A | 32498 | -0.5564 | 0.0781 | 0.001559329 | 50.75 |
| 12 | Breast cancer | rs45631540 | TGGAGGATCACTTGAG | T | 32498 | 0.2565 | 0.0192 | 0.005461817 | 178.46 |
| 13 | Breast cancer | rs78540526 | T | C | 32498 | 0.3033 | 0.0331 | 0.002576983 | 83.96 |
| 14 | Breast cancer | rs9610673 | G | T | 32498 | -0.1081 | 0.0198 | 0.00091636 | 29.81 |

**Table S3** Reverse MR estimates of the associations of between autoimmune diseases and breast cancer using inverse variance weighted

| **Exposure** | **b** | **Se** | **OR** | **95% CI** | **p Value** |
| --- | --- | --- | --- | --- | --- |
| Ankylosing spondylitis | 0.035 | 0.024 | 1.036 | 0.988-1.085 | 0.140 |
| Celiac disease | -0.108 | 0.069 | 1.125 | 0.983-1.288 | 0.118 |
| Inflammatory bowel disease | 0.106 | 0.043 | 1.112 | 1.022-1.210 | 0.014 |
| Multiple sclerosis | -0.040 | 0.062 | 0.961 | 0.851-1.085 | 0.519 |
| Primary biliary cholangitis | 0.064 | 0.079 | 1.066 | 0.913-1.245 | 0.419 |
| Psoriasis | -0.035 | 0.048 | 0.966 | 0.879-1.061 | 0.473 |
| Rheumatoid arthritis | -0.020 | 0.032 | 0.980 | 0.921-1.044 | 0.529 |
| Systemic lupus erythematosus | 0.065 | 0.063 | 1.067 | 0.943-1.207 | 0.300 |
| Type 1 diabetes | -0.047 | 0.048 | 0.954 | 0.868-1.048 | 0.325 |
| Myasthenia gravis | 0.076 | 0.074 | 1.079 | 0.933-1.247 | 0.304 |

**Table S4** Sensitivity analysis of reverse MR estimates.

| **Outcome** | **Heterogeneity** | | | | **Horizontal pleiotropy** | |
| --- | --- | --- | --- | --- | --- | --- |
|  | **MR Egger** | | **IVW** | |  |  |
|  | **Q statistic** | **p** | **Q statistic** | **p** | **intercept** | **p** |
| Ankylosing spondylitis | NA | NA | 0.428 | 0.513 | NA | NA |
| Celiac disease | 0.434 | 0.510 | 0.714 | 0.700 | 0.026 | 0.690 |
| Inflammatory bowel disease | 0.079 | 0.778 | 2.675 | 0.262 | 0.043 | 0.354 |
| Multiple sclerosis | 0.752 | 0.386 | 1.669 | 0.434 | 0.041 | 0.514 |
| Primary biliary cholangitis | 4.881 | 0.675 | 4.927 | 0.765 | 0.008 | 0.836 |
| Psoriasis | 0.151 | 0.697 | 0.343 | 0.842 | -0.017 | 0.737 |
| Rheumatoid arthritis | 19.42 | 0.079 | 21.22 | 0.069 | 0.016 | 0.312 |
| Systemic lupus erythematosus | 8.009 | 0.533 | 8.403 | 0.590 | 0.015 | 0.546 |
| Type 1 diabetes | 8.525 | 0.743 | 11.43 | 0.575 | 0.034 | 0.114 |
| Myasthenia gravis | 6.259 | 0.903 | 6.314 | 0.934 | 0.007 | 0.818 |


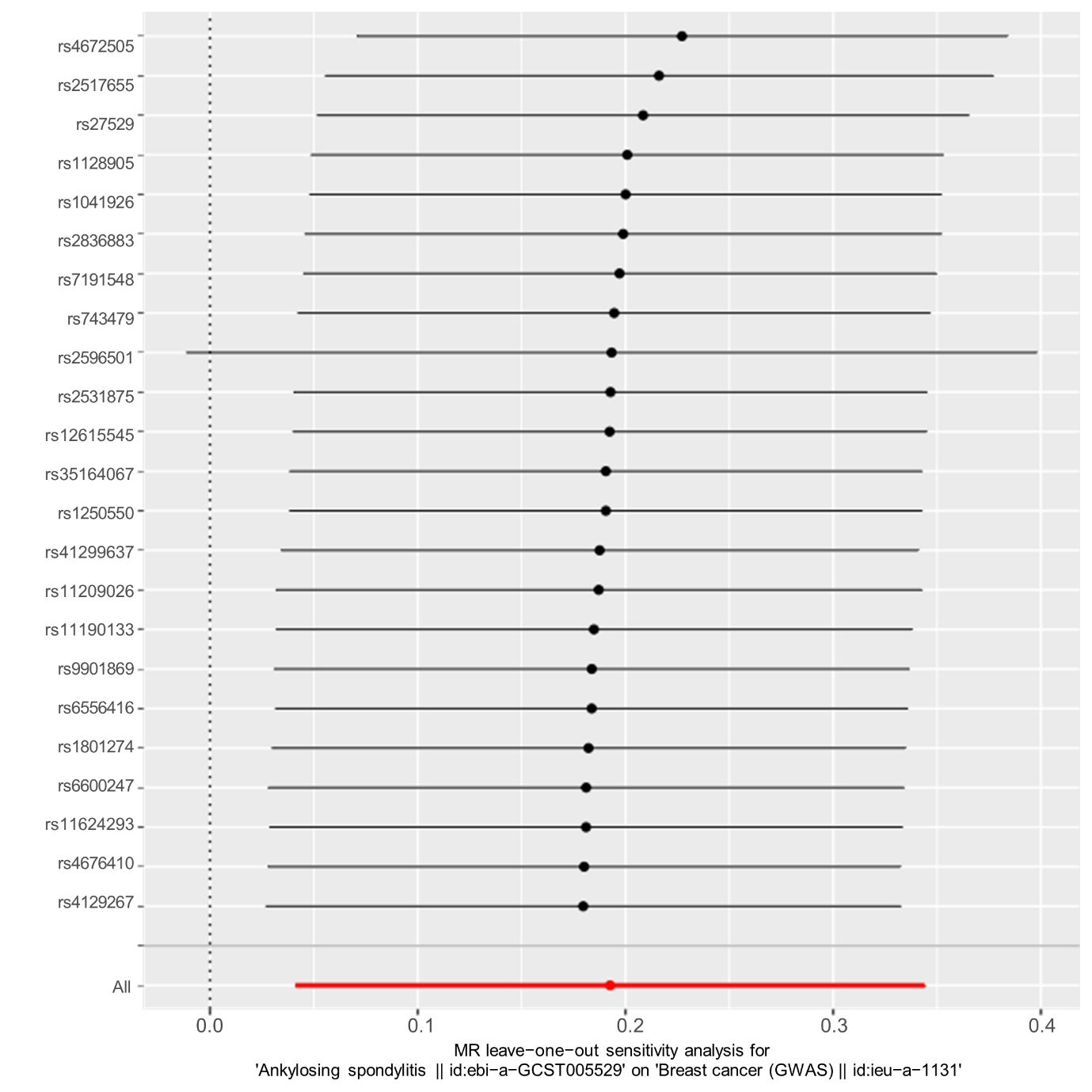


**Figure S1** Leave-one-out analysis of the causal effect of ankylosing spondylitis on breast cancer.


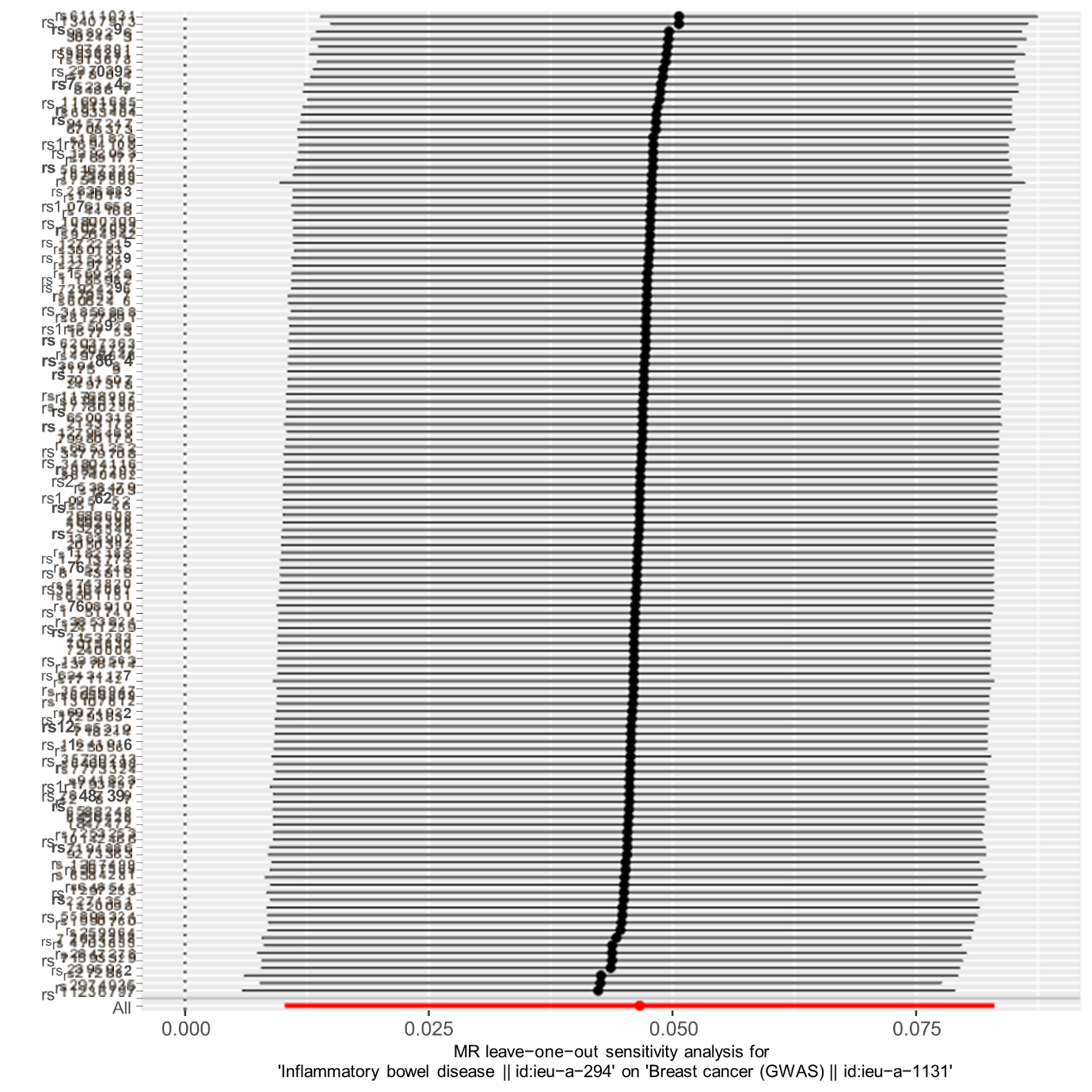


**Figure S2** Leave-one-out analysis of the causal effect of inflammatory bowel disease on breast cancer.


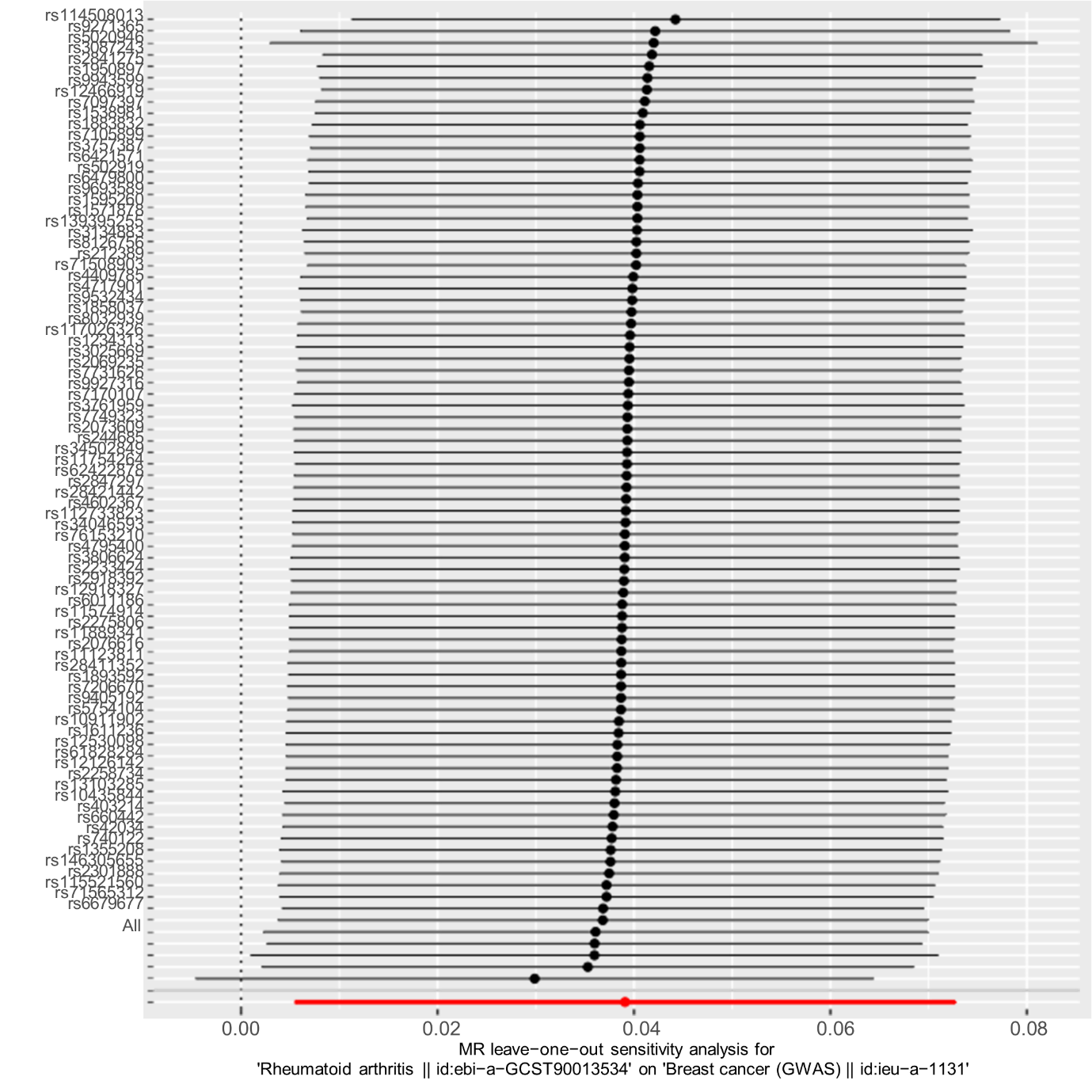


**Figure S3** Leave-one-out analysis of the causal effect of rheumatoid arthritis on breast cancer.


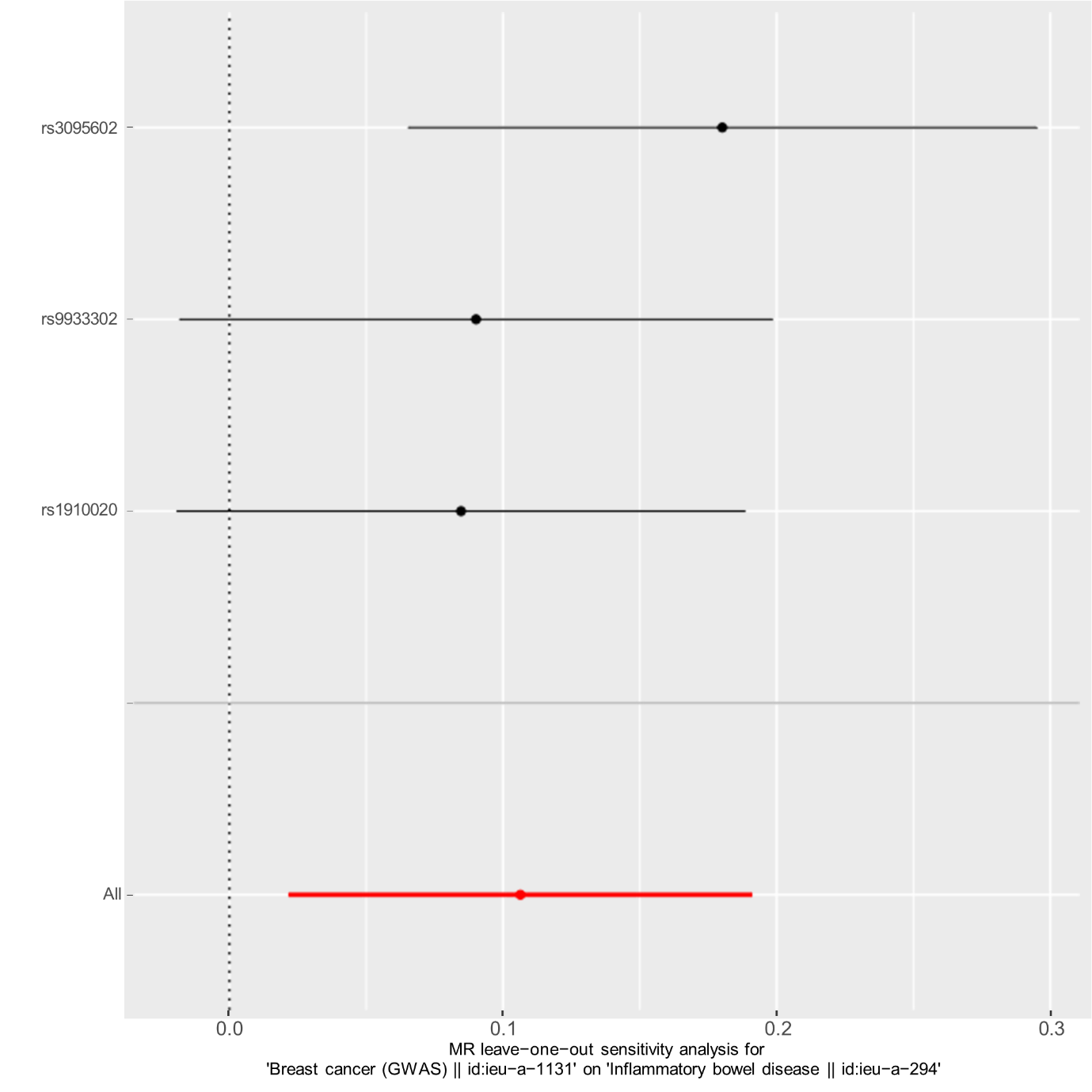


**Figure S4** Leave-one-out analysis of the causal effect of breast cancer on inflammatory bowel disease.
